# Multi‐Metric Approach for the Comparison of Denoising Techniques for Resting‐State fMRI

**DOI:** 10.1002/hbm.70080

**Published:** 2025-05-01

**Authors:** Federica Goffi, Anna Maria Bianchi, Giandomenico Schiena, Paolo Brambilla, Eleonora Maggioni

**Affiliations:** ^1^ Department of Electronics Information and Bioengineering Politecnico di Milano Milan Italy; ^2^ Department of Neurosciences and Mental Health Fondazione IRCCS Ca’ Granda Ospedale Maggiore Policlinico Milan Italy; ^3^ Department of Pathophysiology and Transplantation University of Milan Milan Italy

**Keywords:** benchmarking, denoising pipeline, functional magnetic resonance imaging, preprocessing, resting state

## Abstract

Despite the increasing use of resting‐state functional magnetic resonance imaging (rs‐fMRI) data for studying the spontaneous functional interactions within the brain, the achievement of robust results is often hampered by insufficient data quality and by poor knowledge of the most effective denoising methods. The present study aims to define an appropriate denoising strategy for rs‐fMRI data by proposing a robust framework for the quantitative and comprehensive comparison of the performance of multiple pipelines made available by the newly proposed HALFpipe software. This will ultimately contribute to standardizing rs‐fMRI preprocessing and denoising steps. Fifty‐three participants took part in the study by undergoing a rs‐fMRI session. Synthetic rs‐fMRI data from one subject were also generated. Nine different denoising pipelines were applied in parallel to the minimally preprocessed fMRI data. The comparison was conducted by computing previously proposed and novel metrics that quantify the degree of artifact removal, signal enhancement, and resting‐state network identifiability. A summary performance index, accounting for both noise removal and information preservation, was proposed. The results confirm the performance heterogeneity of different denoising pipelines across the different quality metrics. In both real and synthetic data, the summary performance index favored the denoising strategy including the regression of mean signals from white matter and cerebrospinal fluid brain areas and global signal. This pipeline resulted in the best compromise between artifact removal and preservation of the information on resting‐state networks. Our study provided useful methodological tools and key information on the effectiveness of multiple denoising strategies for rs‐fMRI data. Besides providing a robust comparison approach that could be adapted to other fMRI studies, a suitable denoising pipeline for rs‐fMRI data was identified, which could be used to improve the reproducibility of rs‐fMRI findings.

## Graphical abstract

The present study aims at defining an appropriate denoising strategy for resting‐state fMRI data by quantitatively comparing the performance of multiple pipelines in terms of both artifact removal and signal preservation, including resting‐state network identifiability. This will ultimately contribute to standardizing fMRI denoising steps, reducing analytic flexibility, and improving reproducibility.

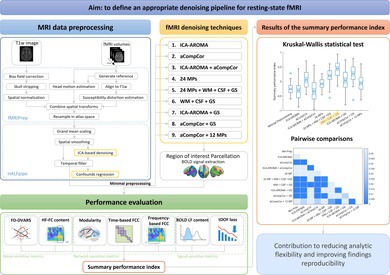

## 1 Introduction

Functional magnetic resonance imaging (fMRI) is a widespread and powerful technique for investigating fundamental properties of brain functional organization and development over the lifespan (Glover 2011).

In recent years, neuroimaging applications have led to an explosion in knowledge about complex human brain functions, which, consequently, has been accompanied by rapid advances in fMRI hardware and software technologies. However, an important problem that affects fMRI data is the presence of artifacts, which can hide, or falsely augment, the hemodynamic and metabolic information embedded in the Blood Oxygenation Level Dependent (BOLD) signal, indirectly related to the neural activity in the brain (Griffanti et al. 2017).

The extraction of meaningful information from fMRI data relies on good data quality, which can be achieved through proper acquisition strategies and by applying adequate preprocessing and denoising procedures to the raw data, and which is essential to guarantee reliable analyses and results.

Hardware‐ and subject‐related artifact identification and removal is a significant problem in task‐based fMRI, but it is even more complex with resting‐state fMRI (rs‐fMRI), where the global state of the brain is of interest; in the absence of an a priori hypothesis, it may be difficult to distinguish the signal related to brain activity from the sources of noise (Tassi et al. 2020). For example, the identification of resting‐state networks (RSNs) and the quantification of resting‐state functional connectivity (FC) can be hindered by the presence of artifacts, since many of them share some spatial or spectral features with RSNs (Soares et al. 2016). Thus, in the definition of the fMRI protocols, one of the main goals is to obtain the highest possible signal‐to‐noise ratio while minimizing the impact of artifacts.

In the last years, a variety of fMRI processing pipelines have been identified and implemented, and the resulting proliferation of software tools designed to fulfill various analytic functions has produced many different options for carrying out any type of fMRI processing, prompting advancement but also sowing confusion. Indeed, a consensus on a standard fMRI processing pipeline has not been reached, as some steps could be optional and with choices to be made about parameters and/or methods. This analytic flexibility, combined with the large number of analysis operations, as well as the number of possible parameters for running each analysis step, has led to a vast multiplicity of methodological variants, possibly leading also to considerable heterogeneity in the results (Poldrack et al. 2017). This situation has contributed to the so‐called *reproducibility crisis* that affects the entire neuroimaging field, including the fMRI one, which has led to an increased demand for standardized workflows to conduct both the preprocessing and processing stages of fMRI analyses (Gorgolewski et al. 2016; Poldrack et al. 2017). The urgent need to develop new practices and tools to overcome the challenge of variability across analysis pipelines and its effect on the results is emphasized also by the findings of a recent study (Botvinik‐Nezer et al. 2020), where the authors assessed the effect of this flexibility on the results obtained by many independent teams that analyzed the same fMRI dataset.

Recently, the ENIGMA (Enhancing Neuro Imaging Genetics through Meta Analysis) Consortium (Thompson et al. 2014) has started to address this problem by developing standardized pipelines for neuroimaging data processing, including but not limited to preprocessing steps, multi‐center harmonization, feature extraction, and quality control. Among the functional image processing protocols, it proposed HALFpipe (Harmonized AnaLysis of Functional MRI pipeline) toolbox, which provides a standardized workflow that encompasses the essential elements of task‐based and rs‐fMRI analyses from raw data to group‐level statistics (Waller et al. 2022). HALFpipe is built on the progresses and contributions of the fMRIPrep software developers (Esteban et al. 2019) and extends its functionality beyond preprocessing steps to include additional processing steps and interactive tools for data quality assessment.

Additionally, HALFpipe software is containerized, meaning that it includes all the software needed for it to run, such as fMRIPrep (Esteban et al. 2019), MRIQC (Esteban et al. 2017), FSL (Jenkinson et al. 2012), ANTs (Avants, Tustison, and Song 2008), FreeSurfer (Fischl 2012), and AFNI (Cox 1996). As such, all users of one version of HALFpipe will be using the exact same versions of these tools (Waller et al. 2022), aiding reproducibility across different researchers' and computing environments (Poldrack, Gorgolewski, and Varoquaux 2019).

However, although HALFpipe provides recommended settings for each of the preprocessing steps (Waller et al. 2022), it allows users to run any combination of the processing steps, thereby complicating the definition of the pipeline to be used and potentially reducing the methodological transparency (Prager et al. 2019). Many different denoising techniques are available to the users, and no standard denoising pipeline is provided and motivated, resulting in a toolbox that is strongly user dependent.

Although all participant‐level confound regression methods aim at mitigating the impact of artifacts on subsequent analysis steps, each of them is more sensitive to noise or signal components identification.

Recently, prior studies have attempted to compare the performance of some existing confound removal strategies on selected benchmark measures, several quality control metrics reflecting different properties of interest (Burgess et al. 2016; Ciric et al. 2017; de Blasi et al. 2020; Hoeppli et al. 2023; Kassinopoulos and Mitsis 2022; Parkes et al. 2018; Scheel et al. 2022; Wang et al. 2024). A common finding in these studies is that the scores obtained from the quality control metrics often yielded contradictory results, for example, pipelines yielding the highest score in terms of RSNs identifiability were found to be less successful in reducing motion artifacts (Ciric et al. 2017).

In this context, the present study aims to define an appropriate denoising procedure for rs‐fMRI data by quantitatively comparing the performance of multiple denoising pipelines based on options (taken separately or in combination) made available by HALFpipe, contributing to standardizing rs‐fMRI preprocessing and denoising steps. Our research also provides a multi‐metric comparison framework that could be adapted to other fMRI applications. The comparison among denoising strategies is achieved by computing the scores of both previously proposed and novel metrics that quantify the degree of artifacts removal, signal of interest enhancement, and RSNs identifiability.

Furthermore, considering the different sensitivity of the different metrics, a summary composite index, which synthesizes multiple metrics into a unified measure, is proposed to capture a valid fMRI denoising pipeline, showing a good trade‐off between artifact removal and signal of interest preservation, which in our application includes RSNs enhancement, and to streamline the interpretation and comparison of the results.

## 2 Methods

### 2.1 Participants

Fifty‐three participants (aged 52.74 ± 21.12 years, 28 females and 25 males) took part in the study. Exclusion criteria for all participants included intelligence quotient < 70, lifetime abuse of alcohol or substances, history of head trauma with loss of consciousness, and neurological or neurodegenerative illnesses. All of them provided written informed consent to the study protocol, which received the approval of the Ethics Committee of the Fondazione IRCCS Ca′ Granda Ospedale Maggiore Policlinico, Milan, Italy and was conducted in accordance with the Declaration of Helsinki.

### 2.2 MRI Data Acquisition

All participants underwent a multimodal magnetic resonance imaging (MRI) session using a 3T Philips Achieva DStream scanner (Philips, Best, The Netherlands) equipped with a 32‐channel head coil. Two hundred fMRI volumes were collected during resting wakefulness with eyes closed using a multi‐transmit T2*‐weighted echo planar imaging (EPI) sequence (field of view (FOV): 120 mm (FH) × 256 mm (AP) × 256 mm (RL), voxel size: 2 × 2 × 2 mm^3^, echo time (TE): 30 ms, repetition time (TR): 2500 ms, flip angle: 90°). Two additional dummy scans were collected. A structural brain image was acquired using a 3D T1‐weighted turbo field echo (TFE) SENSE sequence (FOV: 250 mm (FH) × 240 mm (AP) × 180 mm (RL), voxel size: 1 × 1 × 1 mm^3^, TE: 4 ms, TR: 8 ms, flip angle: 8°) and used as a morphological reference for the fMRI analyses.

The participants were instructed to relax and not to fall asleep, and all of them confirmed to have stayed awake during the session. The resting‐state acquisition was performed after a task‐based fMRI acquisition during a visuomotor Go/NoGo task (Piani et al. 2022).

### 2.3 MRI Data Processing

**FIGURE 1:**
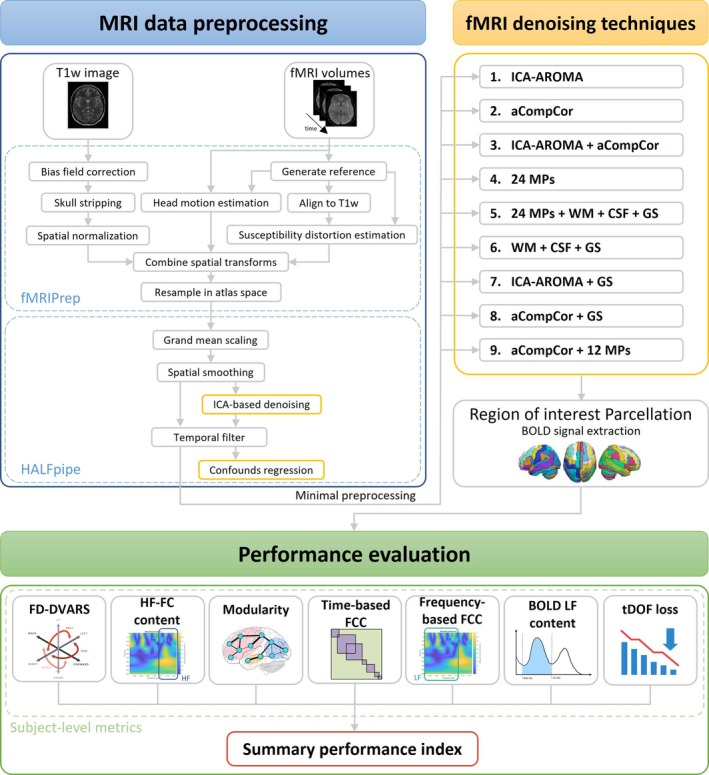
Workflow of the MRI data processing pipeline.

A scheme of the entire MRI data processing stream can be found in Figure 1. The overall workflow is composed of three main blocks, (i) MRI data preprocessing pipeline, (ii) fMRI data denoising pipelines, and (iii) performance evaluation. Raw MRI and fMRI data were preprocessed as presented in the first block, using a set of steps that were shared among all denoising pipelines. Different denoising pipelines were applied in parallel to the preprocessed fMRI data, as shown in the second block. Then, the performances of the different denoising models were compared using quantitative quality control indexes presented in the third block. This workflow was applied to the real rs‐fMRI dataset and to a simulated pilot dataset (Ellis et al. 2020). The simulation study is summarized in Section 2.8, and detailly described in the Supporting Information.

#### 2.3.1 MRI Data Preprocessing

The preprocessing of structural and functional MRI data was performed using HALFpipe (Waller et al. 2022). In HALFpipe, the preprocessing is implemented using fMRIPrep, which performs a consensus of preprocessing steps required for any fMRI study (Esteban et al. 2019). The consensus steps applied in our study included skull stripping, brain tissue segmentation, and spatial normalization of structural images, and motion correction, susceptibility distortion correction, coregistration, and spatial normalization of functional images.

Specifically, spatial normalization of the anatomical image to the MNI template is done through nonlinear registration with antsRegistration (ANTs). The coregistration of the functional image to the corresponding anatomical reference is performed by using boundary‐based linear coregistration, as implemented in Flirt (FSL). For the generation of preprocessed functional images in template space, the following transforms are concatenated: head‐motion parameters, the warping to reverse susceptibility distortions (if calculated), BOLD‐to‐T1w, and T1w‐to‐template mappings. The composition of transforms allows for a single‐interpolation resampling of volumes with antsApplyTransforms (ANTs).

Additional widely used preprocessing steps were applied, including spatial smoothing (FWHM = 6 mm), grand mean scaling, which sets the image mean—defined as the within‐scan mean across all voxels and time points—to a predefined value (grand mean = 10,000), and Gaussian‐weighted temporal filtering (FWHM = 125 s).

In HALFpipe, any filter or transformation applied to the voxel time series is also applied to the nuisance time series. In this way, previously removed variance is not re‐introduced (Hallquist, Hwang, and Luna 2013; Lindquist et al. 2019).

The preprocessed fMRI data are resampled to the standard space MNI, which in fMRIPrep and in HALFpipe corresponds to the *MNI152NLin2009cAsym* template (version 2009c; Horn 2016; Waller et al. 2022).

#### 2.3.2 Data Denoising

Nine different denoising pipelines were parallelly applied to the preprocessed fMRI data to remove residual physiological or exogenous artifacts. All pipelines were implemented in HALFpipe using all the denoising methods made available by the software, considered separately, or combined.

HALFpipe performs fMRI denoising by removing confounds via linear regression, using confounders generated by fMRIPrep, including (i) motion parameters (MPs), (ii) the global signal (GS), which is the mean signal over the whole brain, (iii) the mean white matter (WM) signal, (iv) the mean cerebrospinal fluid (CSF) signal, (v) their principal components, or (vi) noise‐related components calculated from independent component analysis (ICA; Power et al. 2017; Waller et al. 2022).

The following pipelines were implemented.

- *ICA*‐*AROMA*: The pipeline relies on the ICA‐based strategy for Automatic Removal of Motion Artifacts (ICA‐AROMA; Ciric et al. 2017; de Blasi et al. 2020; Hoeppli et al. 2023; Scheel et al. 2022; Wang et al. 2024). It is an ICA‐based technique that consists of an autonomic classification of noise‐ and signal‐related components and the consequential removal of the former. ICA is achieved using MELODIC, part of FSL (Jenkinson et al. 2012). Next, this method uses a predefined classifier that identifies noise‐related components by assessing four features. Specifically, a component is classified as motion‐related when it fulfils at least one of three criteria: (i) exceeding a decision boundary combining the edge fraction and maximum realignment parameter correlation, (ii) a CSF fraction > 10%, or (iii) a high‐frequency content > 35% (Pruim, Mennes, Buitelaar, et al. 2015; Pruim, Mennes, van Rooij, et al. 2015). The estimated ICA‐AROMA noise components are removed from fMRI data using the nonaggressive option to minimize removing variance that is shared between signal and noise component (Waller et al. 2022). No manual interventions on the label's classification were implemented to avoid any performance dependency on the user's experience.
- *aCompCor*: The anatomical Component based on noise Correction method (aCompCor) (Behzadi et al. 2007) performs denoising for the correction of physiological noise by removing the first five principal components extracted from WM and CSF, which are brain structures in which the fMRI time series are not modulated by neural activity (Ciric et al. 2017; Hoeppli et al. 2023; Kassinopoulos and Mitsis 2022; Muschelli et al. 2014; Wang et al. 2024).
- *ICA*‐*AROMA + aCompCor*: The denoising pipeline combines sequentially ICA‐AROMA and aCompCor.
- *24 MPs*: The pipeline includes the removal via linear regression of the three translational and the three rotational MPs and the relative quadratic terms, their six temporal derivatives and their quadratic terms, resulting in a total of 24 MPs regressors (Ciric et al. 2017; Kassinopoulos and Mitsis 2022; Parkes et al. 2018; Satterthwaite et al. 2013).
- *24 MPs + WM + CSF + GS*: The denoising pipeline includes the removal via linear regression of the 24 MPs, the WM and CSF signals and GS (Ciric et al. 2017; Kassinopoulos and Mitsis 2022; Scheel et al. 2022; Wang et al. 2024).
- *WM + CSF + GS*: The pipeline consists of the removal via linear regression of WM, CSF, and GS time series.
- *ICA‐AROMA + GS*: The pipeline combines ICA‐AROMA with GS regression (Ciric et al. 2017; Wang et al. 2023).
- *aCompCor + GS*: The pipeline combines aCompCor with GS regression.
- *aCompCor + 12 MPs*: The pipeline relies on aCompCor denoising with in addition the removal via linear regression of 12 MPs, which are the six original motion parameters and their six temporal derivatives (Muschelli et al. 2014; Parkes et al. 2018).

### 2.4 Atlas‐Based BOLD Signal Extraction

The preprocessed fMRI images resulting from the different denoising pipelines were parceled according to the Automated Anatomical Labeling (AAL) atlas (Tzourio‐Mazoyer et al. 2002). The AAL atlas was selected for these analyses due to its widespread use and validation in the neuroimaging field. It offers a well‐established and standardized set of brain regions that facilitates comparison with the existing literature. While newer atlases with more refined parcellations are available, the AAL atlas provides a balanced number of regions of interest (ROIs), with well‐defined anatomical relevance, making it suitable for large‐scale network analyses without the added complexity of finer subdivisions.

BOLD signal extraction was performed by computing the mean signal of the voxels belonging to the same ROI. The ROIs belonging to cerebellum and vermis were excluded from the analysis because they were not covered in the fMRI volumes of some participants. Hence, we considered the first 90 ROIs of the AAL atlas.

ROI‐level BOLD signals were used to compute the ROI‐to‐ROI FC based on the time‐domain Pearson correlation (t‐FC) and on the time‐frequency‐domain wavelet coherence. The latter spectrum was divided into two portions according to low frequency (0.01–0.1 Hz, LF‐FC) and high frequency (> 0.1 Hz, HF‐FC) ranges that mainly reflect brain‐related and noise‐related processes, respectively.

The extracted BOLD time series and the ROI‐to‐ROI FC measures were used for computing some of the following quality metrics.

### 2.5 Performance Metrics

The nine denoising methods were compared among themselves and with the standard preprocessing using a combination of previously proposed and novel fMRI data quality metrics. The use of multiple metrics allowed us to evaluate the pipeline performances in terms of noise reduction (noise‐sensitive metrics) and signal of interest preservation or enhancement (signal‐sensitive metrics), including RSNs identifiability (network‐sensitive metrics).

All data quality metrics were computed for each subject and for each pipeline, resulting in 530 performance values for each metric. In the following, we describe the metrics used in the present work.

#### 2.5.1 Noise‐Sensitive Metrics

- *FD‐DVARS*: This noise‐sensitive metric is calculated as the Pearson's correlation coefficient between two framewise data quality indices: the framewise displacement (FD) and the derivative of root‐mean‐square variance over voxels (DVARS) (Kassinopoulos and Mitsis 2022).
  FD is a time series that reflects the extent of motion during the acquisition and is defined as the sum of absolute values of the first derivative of the six MPs, after converting the rotational parameters to translational displacements on a sphere of 50 mm radius.
  DVARS is defined as the spatial root‐mean‐square of the voxel time series after they are temporally differentiated. It measures how much the intensity of an fMRI volume varies at each timepoint compared to the previous point (Power et al. 2012). FD depends only on realignment parameters and is unique for each subject's fMRI data, whereas DVARS is based on BOLD signal intensities and is computed for each denoising method separately; from their correlation, it is thus possible to quantify the presence of residual motion in the denoised fMRI data, and hence to evaluate the capability of that pipeline in reducing motion‐related artifacts.
  Moreover, the correlation between the mean FD value of each subject and FD‐DVARS was computed to assess the presence of motion‐related biases. Indeed, the final goal is to obtain FD‐DVARS values that are not influenced by the degree of motion to have low‐motion and high‐motion scans with similar behaviors (Kassinopoulos and Mitsis 2022).
- *HF*‐*FC content*: This noise‐sensitive metric evaluates the HF‐FC content, which is mainly associated with non‐neural sources. For each pair of ROIs, the difference in HF‐FC spectra between pre‐ (minimal preprocessing) and post‐denoising was computed. Then, the mean value over time and frequency of the difference matrix was computed for each pair of ROIs, obtaining a symmetric matrix. The mean value of the matrix upper triangle was derived and used as the metric value. The higher the metric value (i.e., higher differences between pre‐ and post‐denoising), the higher should be the ability of the pipeline to destroy ROI‐to‐ROI connections that are mainly related to noise.

#### 2.5.2 Network‐Sensitive Metrics

**FIGURE 2:**
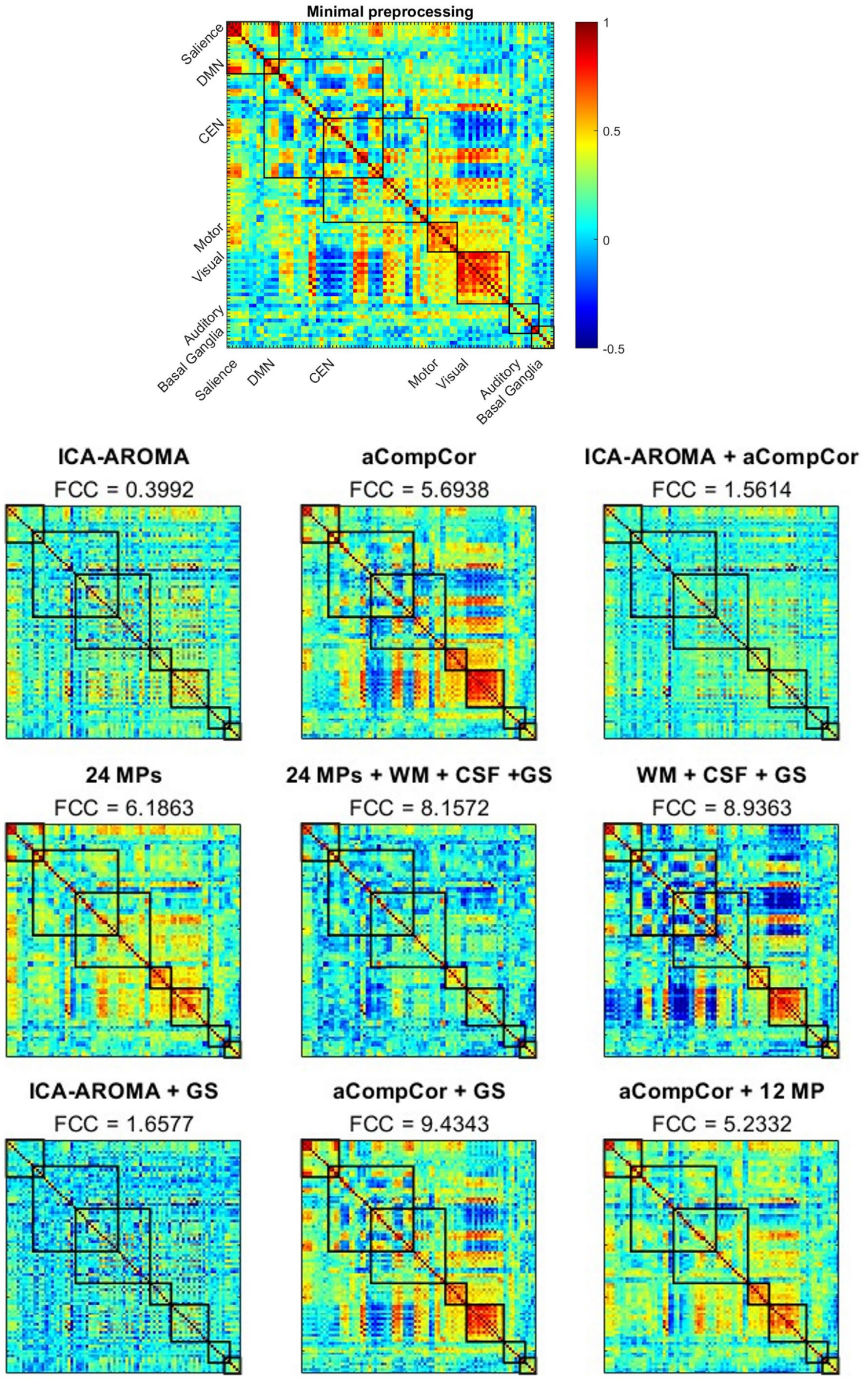
Exemplar multi‐network time‐based FCC. In the figure, the t‐FC matrices and the corresponding time‐based FCC values for the minimal preprocessing (first row, center) and for the different denoising pipelines are reported for an exemplar subject. The ROI‐to‐ROI connections are reorganized in RSNs as reported for the first matrix relative to the minimal preprocessing pipeline. Basal Ganglia = basal ganglia network; CEN = central executive network; DMN = default mode network; Motor = Sensory motor network; Salience = salience network; Visual = visual network; Auditory = auditory network.

- *Modularity*: The network‐sensitive modularity (Q) quality metric was computed on the t‐FC matrix and provides a quantification of the degree to which there are structured sub‐networks in the entire brain network. Q was computed by performing community detection according to the Louvain heuristic, which partitions the connectome into sub‐networks in a way that aims at maximizing the value of Q itself (Blondel et al. 2008; Rubinov and Sporns 2010). This metric can provide an estimate of the degree of enhancement of the intrinsic brain network's modular structures after denoising (Bassett et al. 2011; Betzel, Medaglia, and Bassett 2018; Betzel and Bassett 2017; Puxeddu et al. 2020; Sporns and Betzel 2016).
  In addition, also the correlation between Q and the mean FD of each subject was computed across the different denoising pipelines to evaluate the presence of possible biases in networks identifiability between low‐motion and high‐motion fMRI acquisitions (Ciric et al. 2017).
- *Time*‐*based FCC*: The time‐based functional connectivity contrast (FCC) is a network‐based metric that was computed on the t‐FC. All t‐FC pairwise correlation values were reorganized in RSNs within the matrix, as shown in Figure 2. The assumption over this metric is that on average a pair of parcels belonging to the same network should exhibit a higher correlation value (within‐network edge, WNE) compared to a pair of parcels from different networks (between‐networks edge, BNE). We assumed that if a pipeline improves the signal‐to‐noise ratio in the data, it should also lead to an increase in the difference between t‐FC values corresponding to WNEs and BNEs. FCC was computed as the Z‐statistic of the Wilcoxon rank‐sum test related to the null hypothesis that WNEs and BNEs in the t‐FC are samples from continuous distributions with equal medians (Kassinopoulos and Mitsis 2022). Furthermore, the identifiability of each of the networks separately was computed by considering only the WNEs belonging to the network of interest (rather than WNEs from all networks) when comparing WNEs and BNEs (Kassinopoulos and Mitsis 2022).
- *Frequency*‐*based FCC*: This network‐sensitive metric assesses the capability of identifying the RSNs by focusing on the LF content in the BOLD signals. For each pair of ROIs, the difference of the LF‐FC spectra between pre (minimal preprocessing) and post‐denoising was computed. Then, the mean value over time and frequency of the difference matrix was computed for each pair of ROIs, obtaining a symmetric pre versus post LF‐FC matrix. Using the same pipeline described for the time‐based FCC, the frequency‐based FCC was computed from the pre versus post LF‐FC matrix. We assumed that lower pre versus post differences in the WNEs would correspond to greater increases in the frequency‐based FCC, suggesting greater ability of the pipeline to preserve RSNs connections.

#### 2.5.3 Signal‐Sensitive Metrics

- *Low*‐*frequency BOLD content*: This metric is sensitive to the quality of BOLD signals. Under the assumption that most of the BOLD LF content is related to neural processes, this metric aims at quantifying how much of the frequency content of the signal of interest has been maintained during the denoising phase. The power spectral density was computed through the periodogram for each ROI‐level BOLD signal and for each pipeline. Then, after estimating the LF power (0.01–0.1 Hz), the fraction of LF power remaining after denoising with respect to the LF power before denoising was calculated. The higher the ratio, the higher the preservation of the BOLD signal in the frequency range of the brain activity.
- *Temporal degree of freedom* (*tDOF*) *loss*: This signal‐sensitive metric quantifies the risk of information loss caused by the denoising phase. The total number of fMRI volumes was considered as the total number of available tDOF and each regressor removed from the data was considered as a lost tDOF. The total loss of tDOF was expressed as a percentage of the initial number of available tDOF. Consequently, the lower number of confounds regressed out, the lower the number of tDOF removed, and hence the lower the risk of losing signal of interest (Ciric et al. 2017; de Blasi et al. 2020; Parkes et al. 2018).

An explorative analysis of the relationships among the different quality measures was performed to examine their correlations within and between the three categories and how they relate and complement each other, providing a quantitative rationale for the definition of a summary performance index. A detailed description of the present cross‐correlation analysis is described in the Supporting Information.

### 2.6 Summary Performance Index

The quality metrics described above were combined to achieve a unique composite index that can represent the trade‐off of each denoising pipeline between noise removal and signal of interest preservation. FD‐DVARS and tDOF loss measures, which were in the 0–1 range, were inverted by computing 1 − abs(*x*), where *x* is the score of each metric, so that, similarly to the other metrics, a high positive score is assigned to good quality data. Subsequently, each metric was normalized by subtracting the metric mean and then dividing by its standard deviation.

The normalized metrics were then used to compute a summary quality index, as follows. The mean values of the noise‐sensitive metrics, network‐sensitive metrics, and signal‐sensitive metrics were separately computed. Then, the summary performance index was computed as the mean value of the above three mean values. The formulas used to compute the summary performance index are reported in the Supporting Information.

Two other versions of the summary index were implemented (i) using the geometric mean to aggregate the single quality measures, and (ii) balancing noise and signal by aggregating network‐ and signal‐sensitive measures. These additional versions of the summary index are described in the Supporting Information.

### 2.7 Statistical Analysis

The effectiveness of the different denoising pipelines (including the standard preprocessing) was compared in terms of both single indices and summary performance index by applying the nonparametric Kruskal–Wallis (KW) test. The KW multiple comparison test was followed by pairwise comparisons. The significance threshold was set to *p*‐value = 0.005 to account for the parallel comparisons of eight different performance metrics (Bonferroni correction of *p* = 0.05 considering 8 comparisons), and to maintain a stringent criterion for statistical significance.

### 2.8 Simulation Study

A simulation pilot study was conducted to test the efficacy of the different denoising methods on synthetic rs‐fMRI data. In our study, fMRI data simulation was performed by using fmrisim, which is a package for standardized, realistic simulation of fMRI data (Ellis et al. 2020). For fMRI noise generation, fmrisim toolbox employs a linear model that estimates and combines known sources of fMRI noise, including drift, physiological, and system noise. Instead, rs‐fMRI signal was simulated independently, by generating time series that mimic neuronal activity within the default mode network (DMN). Subsequently, we combined noise and signal components to achieve the entire 4D rs‐fMRI simulation. The entire pipeline defined for real rs‐fMRI data was then applied to the synthetic dataset. More details on the simulation methodology are available in the Supporting Information.

## 3 Results

**FIGURE 3:**
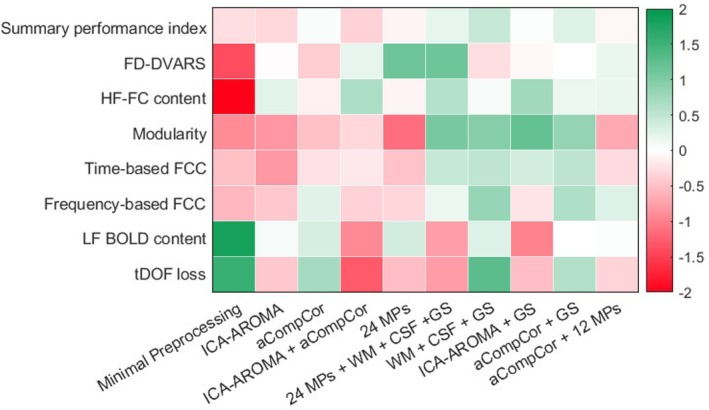
The median values (*z*‐scores) of all performance metrics for the different denoising pipelines are graphically reported. The values are in arbitrary units.

In this section, the main results achieved from the comparison among different denoising strategies for rs‐fMRI are presented according to the types of performance metrics. Then, the results of the statistical comparison in terms of the summary performance index will be shown. The median, and the first and third quartiles of all performance metrics relative to the different denoising pipelines and the corresponding KW statistics are listed in Table 1. The same median values are reported graphically in Figure 3. The post hoc pairwise comparison results are illustrated in Figures S4–S14.

**TABLE 1:** Results of the performance metrics for each denoising pipeline.

Performance metrics	Denoising pipelines (first quartile, median, third quartile)										KW test (*p*‐value)
	Baseline	ICA‐AROMA	aCompCor	ICA‐AROMA + aCompCor	24 MPs	24 MPs + WM + CSF + GS	WM + CSF + GS	ICA‐AROMA + GS	aCompCor + GS	aCompCor + 12 MPs	
FD‐DVARS	−2.099	−0.515	−0.903	−0.305	1.071	1.018	−1.114	−0.468	−0.858	−0.346	1.361e−43
	**−1.405**	**−0.029**	**−0.389**	**0.188**	**1.150**	**1.133**	**−0.265**	**−0.048**	**0.012**	**0.156**	
	−0.486	0.301	0.044	0.588	1.278	1.278	0.496	0.470	0.555	0.613	
HF‐FC content		−0.066	−0.311	0.445	−0.296	0.322	−0.519	0.520	−0.626	−0.174	2.539e−50
	**−2.401**	**0.226**	**−0.111**	**0.641**	**−0.083**	**0.592**	**0.078**	**0.740**	**0.145**	**0.168**	
		0.658	0.514	0.949	0.126	0.837	0.329	1.074	0.487	0.457	
Modularity	−1.297	−1.261	−0.823	−0.869	−1.345	0.833	0.618	0.905	0.253	−0.969	2.288e−60
	**−0.892**	**−0.816**	**−0.494**	**−0.304**	**−1.136**	**1.051**	**0.940**	**1.219**	**0.831**	**−0.663**	
	−0.452	−0.379	0.212	0.235	−0.812	1.290	1.239	1.532	1.243	−0.039	
Time‐based FCC	−0.989	−1.266	−0.708	−0.898	−1.082	0.074	−0.129	−0.107	−0.137	−0.851	9.888e‐19
	**−0.487**	**−0.797**	**−0.232**	**−0.185**	**−0.479**	**0.450**	**0.512**	**0.352**	**0.527**	**−0.294**	
	−0.029	−1.01	0.732	0.349	0.070	1.147	1.006	1.041	1.053	0.794	
Frequency‐based FCC		−1.083	−0.915	−1.049	−0.784	−0.486	0.242	−0.613	−0.057	−0.495	1.208e−9
	**−0.560**	**−0.427**	**0.242**	**−0.346**	**−0.327**	**0.144**	**0.824**	**−0.208**	**0.624**	**0.278**	
		0.299	1.018	0.556	0.165	0.623	1.384	0.626	0.966	0.753	
LF BOLD content		−0.608	−0.412	−1.295	−0.214	−1.198	−0.196	−1.301	−0.583	−0.653	9.942e−54
	**1.822**	**0.069**	**0.318**	**−0.908**	**0.356**	**−0.754**	**0.267**	**−0.982**	**−0.006**	**0.030**	
		0.484	0.871	−0.457	0.634	−0.362	0.742	−0.456	0.692	0.983	
tDOF loss		−0.966		−1.826				−1.052			2.641e−94
	**1.550**	**−0.428**	**0.690**	**−1.288**	**−0.514**	**−0.772**	**1.292**	**−0.514**	**0.604**	**−0.342**	
		0.002		−0.858				−0.084			
Summary performance index	−0.372	−0.562	−0.189	−0.603	−0.197	0.068	0.210	−0.196	0.069	−0.263	1.316e−40
	**−0.257**	**−0.309**	**0.054**	**−0.344**	**−0.086**	**0.198**	**0.448**	**0.039**	**0.265**	**−0.063**	
	−0.134	−0.142	0.308	−0.030	0.061	0.366	0.627	0.313	0.499	0.141	

*Note:* Results of the performance metrics for each denoising pipeline after *z*‐score normalization. The first quartile, the median (in bold), and the third quartile of all performance metrics relative to the different pipelines and the corresponding KW statistics are listed.

The boxplots of the values of the various quality measures (raw, inverted, and normalized values) have been included in the Supporting Information to show the ranges and the distributions of the values during the different steps. Results on the effects of denoising on inter‐individual variability are described in Table S1.

Additional supporting data and figures were included in the Supporting Information, visually demonstrating the effect of the denoising pipelines on data from low‐motion and high‐motion participants.

### 3.1 Noise‐Sensitive Metrics

The noise‐sensitive metrics (i.e., FD‐DVARS and HF‐FC content) showed that denoising models removing a higher number of regressors were more effective in noise removal than models with a lower number of time series regressed out.

Based on the FD‐DVARS metric, the pipelines that resulted more effectively in motion‐related artifacts removal, and hence showed the lowest residual motion content, are those that include the regression of the motion parameters (24 MPs and 24 MPs + WM + CSF + GS). The KW test among all different pipelines showed significant FD‐DVARS differences (*p* < 0.05, Bonferroni corrected); the subsequent multiple pairwise comparisons showed that all the pipelines except for aCompCor were significantly more effective than the baseline preprocessing in removing artifacts related to head movements. The 24 MPs and 24 MPs + WM + CSF + GS pipelines performed significantly better than all the others.

Considering the correlation between FD and FD‐DVARS, among the two top‐performing methods, 24 MPs showed a low but significant correlation value (*r* = 0.304, *p* < 0.05) suggesting different performances in low and high motion scans, instead FD and FD‐DVARS were not significantly correlated in 24 MPs + WM + CSF + GS (*r* = 0.165, *p* = 0.237). Other denoising techniques (aCompCor, ICA‐AROMA + aCompCor, WM + CSF + GS, and aCompCor + GS) showed a not significant correlation value. In these pipelines, high‐motion scans have FD‐DVARS values that are similar to the ones of low‐motion scans, meaning that the removal of movement artifacts is independent of the level of motion during the acquisition. The correlation values between FD and FD‐DVARS and corresponding p‐values are reported in Table S2 for all pipelines.

The comparison based on the HF‐FC content, which is sensitive to the removal of both motion‐related noise and artifacts coming from other sources, showed good performances of all the denoising pipelines. The KW test among all pipelines showed significant HF‐FC content differences (*p* < 0.05, Bonferroni corrected); specifically, all the denoising pipelines were able to remove HF noise that remained after the baseline preprocessing, leading to HF‐FC values that were significantly higher than in the baseline preprocessing; models that contain ICA‐AROMA technique (ICA‐AROMA, ICA‐AROMA + aCompCor, and ICA‐AROMA + GS) and 24 MPs + WM + CSF + GS were the most effective in HF noise removal.

### 3.2 Network‐Sensitive Metrics

The metrics related to large‐scale network identifiability showed consistent results among them. Specifically, the pipelines involving global signal regression (GSR) resulted to be more able to highlight sub‐networks in the overall connectome.

As regards Q, generally regarded as a key feature of biological network organization (Alcalá‐Corona et al. 2021; Satterthwaite et al. 2012), the KW test showed significant differences among denoising pipelines (*p* < 0.05, Bonferroni corrected). The pipelines performing GSR (24 MPs + WM + CSF + GS, ICA‐AROMA + GS, WM + CSF + GS, and aCompCor + GS) showed significantly higher values of Q with respect to the baseline preprocessing and the other denoising methods, meaning that they were more able to identify structured functional subnetworks within the entire brain. Instead, the pipelines that do not perform GSR were comparable to the baseline preprocessing.

The presence of possible biases due to different motion degrees was evaluated by computing the correlation between Q and the mean FD of each subject across the different pipelines. We found low levels of correlation between the two considered variables (all *p* values > 0.05), except for 24 MPs + WM + CSF + GS, which showed a significant correlation between FD and Q (*r* = −0.30, *p* = 0.029), suggesting high Q values when FD is low, with respect to the other techniques which were almost independent on the degree of motion (i.e., a correlation value between mean FD and Q that is close to zero). The baseline processing showed a correlation value close to zero (*r* = −0.0078, *p* = 0.956) meaning that *Q* values are not biased by the level of motion; however, *Q* values in the minimal preprocessing are significantly lower than the ones in the other pipelines. The correlation values between FD and *Q* and corresponding *p* values are reported in Table S3 for all pipelines.

As regards time‐based FCC, which aims at quantifying how much the RSNs are highlighted with respect to the overall background activity, the KW test showed significant results (*p* < 0.05, Bonferroni corrected), and the following pairwise comparisons revealed significant differences between the minimal preprocessing and the denoising pipelines that perform GSR, confirming the results of modularity metric. The other denoising techniques were nonsignificantly different from the minimal preprocessing in terms of FCC. The network‐specific FCC values, reflecting the identifiability of each RSN separately, for all denoising pipelines are illustrated in Figure S28.

A development of time‐based FCC is frequency‐based FCC, which has been more specifically optimized to capture inter‐network correlations mainly related to neural activity. The frequency‐based FCC revealed significant differences among the pipelines based on the KW test (*p* < 0.05, Bonferroni corrected). Denoising techniques performing WM + CSF + GS and aCompCor + GS were the most able to highlight RSNs in the LF range with respect to the background connections, resulting significantly different from the minimal preprocessing, whereas all the other models resulted comparable to the minimal preprocessing.

As for the above metrics, for frequency‐based FCC, the best‐performing pipelines involve GSR. This result agrees with the two previous network‐related metrics. In addition, the frequency‐based FCC metric showed that WM + CSF + GS and aCompCor + GS pipelines were more conservative on the information of interest (preserving LF FC links) than 24 MPs + WM + CSF and ICA‐AROMA + GS, which, regarding to network sensitivity, were rewarded only by modularity and time‐based FCC metrics.

### 3.3 Signal‐Sensitive Metrics

The KW test applied to the LF BOLD content metric showed significant differences among the pipelines (*p* < 0.05, Bonferroni corrected). The post hoc pairwise comparison showed that all the denoising pipelines removed a portion of LF content in the BOLD signals, since they were significantly different from the baseline preprocessing. ICA‐AROMA + aCompCor, ICA‐AROMA + GS, and 24 MPs + WM + CSF + GS resulted in removing the highest amount of LF BOLD oscillations. Instead, pipelines that included aCompCor (aCompCor, aCompCor + GS and aCompCor +12 MPs), WM + CSF + GS, ICA‐AROMA and 24 MPs were those that better preserved fluctuations in the frequency range of interest.

The comparison based on the tDOF loss metric, which counts how many time series are regressed out during the denoising phase, showed similar results. The KW test showed significant differences among the different pipelines (*p* < 0.05, Bonferroni corrected), with the majority of denoising pipelines associated with significantly higher tDOF loss values compared to the baseline preprocessing. The only denoising pipeline that was not significantly different from the baseline preprocessing was WM + CSF + GS.

### 3.4 Summary Performance Index

The results of the previously defined metrics were equally combined to identify which pipeline could be the more effective, simultaneously, in noise and artifacts removal, networks identifiability and signal of interest preservation.

**FIGURE 4:**
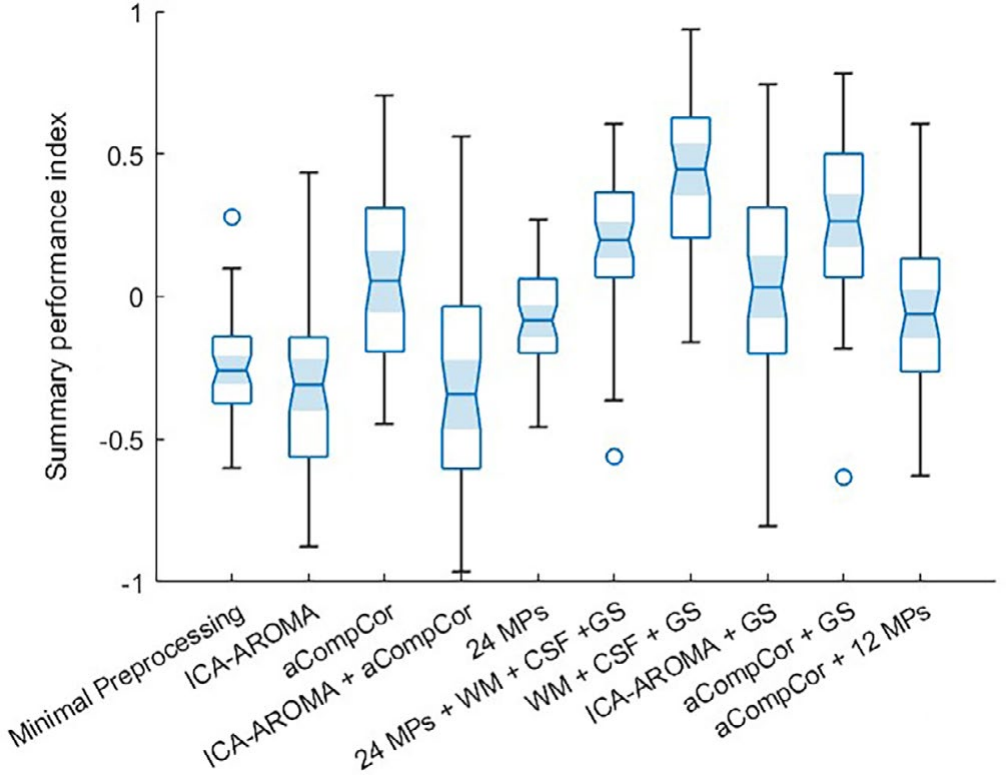
Results of the multiple comparison of the different denoising pipelines according to the summary performance index. The boxes represent the interquartile range, which spans from the 25th percentile to the 75th percentile of the data. The line inside the box indicates the median value.

The KW comparison based on the summary index reported significant differences among pipelines (*p* < 0.05, Bonferroni corrected). The boxplots relative to the summary performance index for all denoising pipelines are reported in Figure 4. The post hoc multiple comparisons showed that the denoising method with WM + CSF + GS regression outperformed the other denoising techniques. This pipeline performed better than the baseline preprocessing, ICA‐AROMA, ICA‐AROMA + aCompCor, ICA‐AROMA + GS, 24 MPs, aCompCor and aCompCor +12 MPs, which were associated with significantly lower values of the summary performance index, and it resulted comparable to 24 MPs + WM + CSF + GS and to aCompCor + GS.

### 3.5 Simulation Study Results

The explorative results obtained on the pilot synthetic rs‐fMRI dataset are consistent with those from the real rs‐fMRI data. The pipelines including ICA‐AROMA provided the highest performance in artifacts removal. Pipelines including GSR showed higher values in network‐sensitive measures. In terms of signal‐sensitive metrics, WM + CSF + GS, aCompCor, and aCompCor + GS provided the best results. The summary performance index indicated that WM + CSF + GS was among the best performing pipelines, followed by pipelines including aCompCor. Additional details on the simulation results are reported in the Supporting Information.

## 4 Discussion

Despite the increasing use of rs‐fMRI data for studying the spontaneous functional interactions within the brain, robust applications of this technique are often hampered by insufficient data quality and by poor knowledge of the most effective preprocessing schemes. In the present study, recently proposed denoising strategies for rs‐fMRI data were quantitatively evaluated according to benchmarks that were selected to capture different domains of effectiveness.

We focused on the comparison of preprocessing denoising techniques that are available in HALFpipe (Waller et al. 2022). Although this tool is being increasingly used, it offers many different denoising options, making its usage strongly user‐dependent and less reproducible than initially expected.

The included benchmarking measures are metrics that are sensitive to (i) the residual noise within fMRI data, (ii) the preservation of the signal of interest, and (iii) the capability to detect RSNs. A combination of these factors was included in a composite performance index proposed to summarize the results. Based on the latter metric, our findings on both real rs‐fMRI data and simulated data support the use of WM + CSF + GS regression denoising strategy, which in our application led to the best compromise between the amount of removed artifacts and the preservation of the information on RSNs.

### 4.1 Comparison With Previous Studies

Until now, only few rs‐fMRI studies evaluated the performance of different denoising pipelines, providing results that need replication and validation (Burgess et al. 2016; Ciric et al. 2017; de Blasi et al. 2020; Hoeppli et al. 2023; Kassinopoulos and Mitsis 2022; Parkes et al. 2018; Scheel et al. 2022; Wang et al. 2024). As a result, there is still no consensus on the optimal strategy, expanding the *reproducibility crisis* of the neuroimaging field and reducing the opportunity of having robust and comparable results among different studies and laboratories.

Specifically, the previous studies, which are the main references for denoising benchmarks, focused on evaluating motion artifact correction (Ciric et al. 2017; Parkes et al. 2018) and its influence on FC measures (Kassinopoulos and Mitsis 2022), leaving out the assessment of the removal of other types of artifacts and a specific quantification of signal of interest preservation.

Another work focused on the comparison of noise‐regression techniques in a large cohort of older adults, considering that motion and physiological noise characteristics may differ substantially for this population (Scheel et al. 2022). A different approach was conducted in the study by Hoeppli et al. (2023), in which the comparison of five preprocessing protocols has been performed quantitatively assessing brain activations in regions of interest, associated with a series of stimuli proposed during task‐based fMRI recordings. Notably, a recent study by Wang et al. (2024) presented a fully reproducible denoising benchmark featuring a range of denoising strategies and evaluation metrics, built primarily on fMRIPrep software.

The present work fits into this context, addressing the problem from different points of view that are particularly valuable in rs‐fMRI studies. Notably, for the first time, our study provides useful knowledge for the selection of the most suitable denoising pipeline among the different options offered by HALFpipe, which is expected to become the pre‐elaboration and elaboration toolbox of reference for functional neuroimaging applications.

With respect to previous studies, a multi‐perspective approach has been adopted by implementing novel performance measures (HF‐FC content and LF BOLD content) and improving other existing ones (frequency‐based FCC). Of note, FCC measure proposed by Kassinopoulos and Mitsis (2022) has been evolved to be more sensitive to oscillations in the BOLD signal frequency range that is usually assumed as related to neuronal activity processes, ignoring spectral power components that could come primarily from other confounding sources.

Taken together, the presented results confirm the performance heterogeneity of commonly used denoising strategies across different quality control metrics that was already underlined in previous benchmarking works (Ciric et al. 2017; de Blasi et al. 2020; Hoeppli et al. 2023; Kassinopoulos and Mitsis 2022; Parkes et al. 2018; Scheel et al. 2022; Wang et al. 2024). In selecting among denoising methods, our results remark on the importance of being aware of the relative strengths and weaknesses of each approach, and of understanding how the preprocessing strategy may impact on the findings in order to identify the most valuable technique according to the research question and the study design (Ciric et al. 2017; Hoeppli et al. 2023).

In this context, our study increased our understanding of shared and distinct characteristics of different denoising strategies, enabling the identification of methods that represent a good compromise between removal of confounders and preservation of rs‐fMRI information. If reproduced on independent datasets, our findings contribute to the ambitious aim of creating a robust rs‐fMRI preprocessing workflow that could be consistently adopted across different research centers.

### 4.2 Main Results

According to the composite performance index proposed by our work, the best performing denoising method for rs‐fMRI applications consists of the regression of the GS and the mean signals from WM and CSF areas.

In contrast with previous evidence, our results suggest that the most valuable denoising methods for rs‐fMRI applications are not necessarily the ones that remove the greatest number of confounding regressors. In our study such approaches appeared to be also the most aggressive on the BOLD signal, hence compromising the preservation of information of interest.

Indeed, as shown by the metric that quantifies the tDOF that are lost during denoising, ICA‐AROMA is one of the costliest methods; and considering the HF‐FC content performance metric, the pipelines including ICA‐AROMA were found to be usually effective in artifact removal, through the elimination of most noise‐related independent components. Nevertheless, in our application, although ICA‐AROMA noise components were removed via regression with the nonaggressive option, these techniques were less conservative than the others on the information of interest, as shown by network‐ and signal‐sensitive measures. Specifically, as shown in Figure 2 for an exemplar subject, t‐FC links within RSNs are decreased in the pipelines including ICA‐AROMA, suggesting that it could have removed signals of interest.

ICA‐AROMA denoising relies on the automatic classification of the extracted components in signal‐related and noise‐related, which sometimes leads to non‐optimal results. This drawback might be partly overcome via a semi‐automatic approach that employs the inspection of spatial maps, time series, and spectral powers of the independent components marked as artifactual, which sometimes include signal‐related components. Based on these considerations, ICA‐based methods are particularly promising for artifact removal, deleting most of artifactual contributions, but further attention should be put in the correct detection and classification of signal‐related and noise‐related components.

In previous studies, ICA‐AROMA has been presented as one of the best strategies that trade off the ability to remove impact of motion and number of tDOF lost and was recommended to add GSR as part of the regressors (Ciric et al. 2017; Parkes et al. 2018). Also, aggressive ICA‐AROMA is likely the most suitable noise regression technique for rs‐fMRI studies of older adults (Scheel et al. 2022), and ICA‐based noise‐reduction techniques have shown to increase signal detection in specific tasks, such as noxious heat stimulation (Hoeppli et al. 2023). However, our results are in line with the findings by Wang et al. (2023, 2024) that supported to avoid the use of ICA‐AROMA (also in combination with GSR) as multiple metrics departed from the conclusions of previous denoising benchmark works.

Furthermore, noise correction strategies that use variable tDOF across participants, as ICA‐based techniques, can lead to artifactual group differences in FC (Yan et al. 2013) and biased group‐level analyses (Ciric et al. 2017). Moreover, methods employing ICA‐AROMA or including the removal of 24 MPs do remove many tDOF compared with other methods. As the number of time points in BOLD signals represents the number of tDOF that are available for statistical inference, fewer residual tDOF can spuriously increase FC (Yan et al. 2013).

Our results showed that simple regression of head MPs, with expansion terms included, is effective in removing motion artifacts from fMRI intensity, as shown by FD‐DVARS results, but it does not seem sufficient to enhance network identifiability with respect to other methods. In this context, we found that adding mean tissue physiological regressors (i.e., WM and CSF signals) and GS improved denoising efficacy, as shown by network‐sensitive metrics. These findings are consistent with past studies, showing that MPs regression is insufficient for removing the movement effects on brain FC (Ciric et al. 2017; Parkes et al. 2018; Yan et al. 2013). The regression of MPs and GS is suggested as the best choice for analysis that requires low loss of tDOF and, also, preserve continuous sampling time series, which is broken by scrubbing (Wang et al. 2024).

Denoising pipelines incorporating variants of aCompCor showed performances similar to those of other strategies. Considering motion artifact reduction, they were comparable to all the other methods except for methods including MPs regression. Regarding other kinds of artifacts, they showed a performance like other denoising pipelines except for the ones including ICA‐AROMA, which instead were more effective in these terms. On the contrary, they seem reliable in signal of interest preservation with respect to other more aggressive methods; and, notably, in our study the combination of aCompCor with GRS has been shown to enhance RSNs.

Benchmark results for aCompCor reported by Ciric et al. (2017) were similar to the results of other denoising models that included GSR, suggesting that while aCompCor does not explicitly include GSR, the practical results of its application are in fact similar. Our work additionally showed that complementing aCompCor with GSR, as for the first time proposed by us, further improved its performance for rs‐fMRI applications since it increased brain network modularity and enabled a clearer RSNs identifiability.

However, Parkes et al. (2018) reported that aCompCor technique is not sufficiently robust since a dependence exists on different levels of motion. Specifically, the authors examined the relationship between motion and FC to assess the efficacy of the various denoising approaches in removing motion‐related variance. Considering the different performance of aCompCor in the acquisitions with low‐level versus high‐level motion, they stated that aCompCor may only be viable in low‐motion data. This result has been consistently found in the recent work (Wang et al. 2023, 2024).

Nevertheless, aCompCor and the other methods removing WM and CSF contributions should be able to remove physiological noise in the absence of additional physiological data. In our study, we could not directly assess the residual physiological noise, but it should be noticed that aCompCor has been validated with RETROICOR denoising technique (Behzadi et al. 2007), which instead uses recordings of the cardiac and respiratory cycles taken during the fMRI scan to model and remove physiological noise (Glover, Li, and Ress 2000). Other strategies for physiological artifact mitigation are based on convolution models that make use of cardiac and respiratory activity recordings for estimating the effect of heart rate (Chang, Cunningham, and Glover 2009) and breathing patterns (Birn et al. 2008). Besides requiring physiological data, these models assume that a linear stationary system can describe these effects, which may not be entirely true (Kassinopoulos and Mitsis 2022). Therefore, by eliminating the need for extra physiological recordings, data‐driven approaches simplify the experimental setup and reduce the complexity of the modeling process and ensure robustness across different populations and conditions.

Despite the low number of confounds regressors, the WM + CSF + GS regression strategy outperformed commonly used models based on MPs or ICA‐AROMA. This suggests that brain tissue signal regressors are informative and able to capture global changes within different brain tissues of no interest, which could be related to head movements or physiological confounds, such as large vessel pulsation (Wagshul, Eide, and Madsen 2011).

This result is in line with the results obtained in the work by Ciric et al. (2017), in which a very simple two‐parameter model (WM, CSF) outperformed commonly used models based on realignment parameters alone (6 or 24 MPs).

In addition, our results support the use of models that utilize GSR. Across nearly every benchmark, the denoising models without the regression of the GS underperformed relatively to similar models that included it. Specifically, as shown by the results of the network‐sensitive measures, the pipelines including GS had higher modularity, time‐based FCC, and LF‐FCC values with respect to the same models without GSR. GSR emerges as a very efficacious strategy for the identification of large‐scale networks in the overall brain network, an aim that is particularly important in rs‐fMRI analyses, where the global state of the functional brain networks is of interest (Tassi et al. 2020). This is in accordance with the results presented in the work by Ciric et al. (2017), where all the top six confound regression approaches included GSR, likely to be the single most efficacious strategy for denoising, and it is further supported by recent data regarding the role of motion and physiological artifacts in GS (Power et al. 2017). The inclusion of GSR increased network modularity also in the denoising strategies considered in the recent work by Wang et al. (2024).

The effectiveness of GSR is probably due to the nature of the motion artifact itself: in‐scanner head motion tends to induce widespread reductions in signal intensity across the entire brain parenchyma (Satterthwaite et al. 2013). This effect is highly reproducible across datasets and is effectively captured by time series regression of the GS (Power et al. 2017).

However, despite its simplicity, the use of GSR within denoising procedures is quite debated (Murphy and Fox 2017). Several studies have shown that a large fraction of the GS is associated with physiological processes such as heart rate and breathing activity (Chang and Glover 2009; Kassinopoulos and Mitsis 2019; Zhu et al. 2015), as well as head motion (Power et al. 2014), but there is also evidence that the GS may be driven by neuronal activity (Fox et al. 2009; Wong et al. 2013). Therefore, even though our results are in support of performing GSR, such a strategy entails the possibility of removing some neuronal‐related fluctuations from the data.

In support of this, it is conceivable that the GS may be strongly correlated with the signals of the most evident RSN. Considering that the DMN showed high glucose metabolism during rest (Buckner, Andrews‐Hanna, and Schacter 2008; Raichle et al. 2001), there could be the hypothesis that the GS may have a high association with DMN dynamics (Chen et al. 2012). For this reason, removing GS could remove neuronal contributions from fMRI signals. In this context, we quantified the identifiability of the most acknowledged RSNs, including the DMN, to evaluate if their specific recognition decreased performing GSR. As shown in Figure S28, our results showed that pipelines including GSR did not reduce DMN identifiability, since time‐based FCC was higher than in the other denoising techniques.

In addition, by shifting the distribution of correlation coefficients to be approximately zero‐centered, GSR could increase the number of negative connections in the functional connectome, and the underestimation of true positives measures could affect the interpretation (Murphy and Fox 2017). Furthermore, it should be noticed that the removal of the GS, which may contain information intrinsically related to the acquisition setting, could help uniform, and harmonize preprocessed fMRI data among different research centers, where different scanners and imaging parameters were used.

Similar considerations can be made for WM signal regression. In the past, the vast majority of fMRI studies have been focused on detecting BOLD changes in brain GM and only a very limited number of reports provided corresponding changes in WM; whereas, in recent years, a growing number of studies have reliably detected BOLD signals within WM which appear to reflect the intrinsic neural activity (Ding et al. 2013, 2016, 2018; Fraser et al. 2012).

Since there is new evidence that even WM has BOLD signal fluctuations that could be task dependent (Gawryluk et al. 2011; Gawryluk, Mazerolle, and D'Arcy 2014; Li et al. 2019; Mazerolle et al. 2010), showing that changes in neural activity are encoded in BOLD variations throughout the brain, there is the risk of regressing out real signal from the data we are analyzing. However, for all the considered denoising pipelines, the possibility of removing neuronal contributions remains a risk of each preprocessing step.

Similarly, caution should be taken for the preprocessing of fMRI data acquired during tasks associated with substantial changes in the WM and in global cerebral blood flow (Coghill et al. 1998; Zeidan et al. 2015). Indeed, as shown in the work by Hoeppli et al. (2023), considerably less brain activation was detected in response to noxious heat stimulation, which is known to elicit correlated WM responses, in data preprocessed with CompCor‐based techniques, probably due to the removal of principal components from WM areas during stimulation that may be associated with systemic physiological responses.

In summary, our results based on complementary perspectives suggest that a single confounder regression strategy might not be optimal for every study considering that the proposed performance measures reward different denoising techniques. Nevertheless, in studies of network organization, such as rs‐fMRI analyses, network identifiability may be of primary interest and, therefore, performing GSR could help enhance the network modularity. This is particularly important in rs‐fMRI applications, where it is more difficult to measure the quality of the data and to preserve information of interest, in the absence of hypotheses on brain regions activations.

The present study provides different valuable options for the analysis of brain networks in resting‐state conditions. Our summary performance index has enabled the identification of a denoising pipeline providing a good compromise between noise removal and information preservation in rs‐fMRI applications.

Overall, our findings aimed to support future fMRI studies in the choice of suitable denoising pipelines; nevertheless, it should be remarked that this choice should be based on the specific interests and characteristics of the individual applications.

### 4.3 Limitations

Some limitations of the present study should be discussed. One of the principal challenges in evaluating the performance of fMRI denoising strategies is the lack of a noise‐free ground truth. The baseline hypothesis is that of removing subject‐ and scanner‐related artifacts within the fMRI data while preserving metabolic and hemodynamic information, which, however, is unknown or difficult to identify.

While this study provides valuable insights into the effectiveness of various denoising techniques, without a ground truth in resting‐state fMRI, it is challenging to establish the absolute reliability of the quality control metrics when comparing denoising techniques.

Some concerns apply to the inclusion of quality metrics targeting the connectivity of RSNs, since these networks have been primarily identified using rs‐fMRI data potentially affected by artifacts; nevertheless, the reliability of fMRI‐based RSNs is supported by electroencephalography (EEG) and EEG‐fMRI evidence (Britz, van de Ville, and Michel 2010; Custo et al. 2017), thus limiting the risk of using biased quality metrics.

Some potential limitations regard the assumptions behind time‐based FCC and HF‐FC content. The LF‐FCC was developed based on the assumption that LF oscillations within RSNs primarily reflect spontaneous neuronal activity, as supported by the widespread use of the Amplitude of Low Frequency Fluctuation (ALFF) index (Yu‐Feng et al. 2007; Zou et al. 2008). However, we recognize that LF physiological noise, such as cardiac and respiratory artifacts, can alias into this range. Therefore, we suggest caution in interpreting frequency‐based FCC results. The HF‐FC content assumed that HF signals predominantly represent noise, but recent studies suggested that brain connectivity may also be present in HF band (DeRamus et al. 2021; Ziaeemehr and Valizadeh 2021). Future research should explore more sophisticated methods for distinguishing between noise and real connectivity in both the LF and HF bands.

Another limitation to be discussed regards the biases introduced by noise‐sensitive quality metrics, like FD‐DVARS, which clearly favored the pipelines that specifically targeted movement artifacts, and signal‐related measures, as tDOF loss that obviously rewarded the pipelines with the lower number of confounding regressors. However, this bias was partly addressed by relying on the summary performance index, which was precisely developed for balancing artifact removal and signal of interest preservation.

Additionally, with regard to the summary performance index, the normalization of metrics with different value ranges could have reduced their sensitivity. Despite that, the results of the single measures can provide crucial additional information on their performance. Within this context, the applicability of the summary index may vary depending on the dataset and research objectives. Therefore, we would like to emphasize the need for researchers to critically assess their data and select appropriate denoising steps based on their specific objectives.

Another limitation is due to a bug in the version of fMRIPrep used by HALFpipe, in which the aCompCor components are currently selected exclusively from the CSF mask (GitHub n.d.). Future versions of HALFpipe are expected to address this bug, which may influence the choice of components in subsequent studies.

Other limitations are related to the dataset used in this study. A possible limitation is in the acquisition protocol. The fact that participants were instructed to keep their eyes closed during fMRI scanning might have affected the FC stability, as recently reported (Patriat et al. 2013). Nevertheless, the literature evidence on brain network differences between closed‐eyes and open‐eyes resting state is contrasting (Han et al. 2023). Although closed‐eyes resting‐state increases the risk of falling asleep, in our study all participants explicitly reported staying awake. In our study, we used a TR of 2.5 s, which is slightly longer than the more commonly applied 2 s in rs‐fMRI studies. Previous research indicated that variations within 0.1–3 s as sampling rates have minimal effects on temporal and FC measures (Huotari et al. 2019). However, the longer TR may increase the risk of aliasing from physiological signals, such as respiratory and cardiac fluctuations, into the LF band of the BOLD signal, potentially affecting the assumptions underlying some quality control measures based on LF and HF ranges, as discussed above. Despite this, the difference between 2 and 2.5 s is relatively small and within an acceptable range for FC studies.

A further limitation of the present study is related to the relatively small dataset available. In the near future, the comparison of denoising pipelines in a replication sample will be useful to assess the reproducibility of the present results. Moreover, we do not know if the present results generalize to datasets with different MRI scanners and sequence parameters, such as much shorter or longer time series or time repetition intervals.

Furthermore, in‐scanner motion itself, as well as physiological noise, could represent a biologically informative phenotype that might be erroneously disregarded. For example, Zeng et al. (2014) found specific alterations in connectivity for participants who had generally high levels of motion, even on scans where motion was low. However, head motion is tracked, hence it is feasible to remove information that is strictly associated with the movements during the acquisition, and correctly dissociate confounding effects of motion on brain connectivity measures.

In addition, it is possible that the characteristics of the different artifacts may be dependent on the population under study. The present study examined the efficacy of denoising strategies in a sample of adults and elders from the general population and did not explore the extent to which the efficacy of each denoising strategy was age‐ or population‐dependent, which merits additional investigation in the future.

Finally, our evaluation included many of the most commonly used techniques among the available strategies for rs‐fMRI denoising, even if other approaches, such as RETROICOR (Glover, Li, and Ress 2000) and ICA‐FIX (Griffanti et al. 2014; Salimi‐Khorshidi et al. 2014), may be valuable. However, in the present study, we focused solely on denoising methods that are available in HALFpipe, which was proposed within ENIGMA consortium for neuroimaging analyses, towards providing a uniform consensus pipeline for rs‐fMRI data preparation.

However, considering the multiple denoising scenarios that can be implemented in HALFpipe, we are aware that the ones compared in our study did not cover all the possible combinations. Future work will involve a comprehensive evaluation of additional denoising pipelines and brain parcellations.

In the future, approaches removing physiological artifacts estimated with modalities other than fMRI, such as heart pulse or respiratory artifacts extracted from electrocardiography or respiration signals (Goffi et al. 2023, 2024) should be included in the comparison.

## 5 Conclusions

The present study provided a comprehensive comparison of the performance of different denoising techniques for rs‐fMRI applications using previously proposed as well as novel quality control metrics, contributing to the ongoing research in this area. Since the metrics had specific sensitivity and did not uniformly favor a specific denoising strategy, showing substantial heterogeneity in the performances, to facilitate the comparison across strategies we adopted a multi‐perspective framework and proposed a summary performance index aiming at identifying a suitable denoising pipeline for rs‐fMRI studies. The investigated measures combined in a unique index rewarded the denoising strategy including the regression of mean signals from WM and CSF brain areas and GS, which showed a good trade‐off between artifacts removal, signal of interest preservation, and RSNs identifiability.

If confirmed on independent datasets, our findings can contribute to the definition of new guidelines for rs‐fMRI denoising, reducing analytic flexibility and improving the reproducibility of rs‐fMRI results.

## Author Contributions

**F.G.:** contributed new analytical tools; analyzed data; writing – original draft. **A.M.B.:** methodology review; writing – review and editing. **G.S.:** performed research; writing – review and editing. **P.B.:** performed research; supervision; writing – review and editing. **E.M.:** designed research; performed research; methodology review; writing – review and editing.

## Ethics Statement

The study protocol received the approval of the Ethics Committee of Fondazione IRCCS Ca′ Granda Ospedale Maggiore Policlinico, Milan, Italy, and was conducted in accordance with the Declaration of Helsinki.

## Consent

All participants provided written informed consent to the study protocol.

## Conflicts of Interest

The authors declare no conflicts of interest.

## Supporting information


**Data S1:** Supplementary Information.

## Data Availability

Raw data were generated at Fondazione IRCCS Ca’ Granda Ospedale Maggiore Policlinico, Milan, Italy. Data supporting the findings of this study are available upon request. The Matlab analysis codes are available at the Open Science Framework web application link https://osf.io/pdc3g/?view_only=ccf4d713fd7a4c7bb5787df9d3103a06.
