# Supplementary material for: Multi‐Metric Approach for the Comparison of Denoising Techniques for Resting‐State fMRI
Source: Hum Brain Mapp. 2025 May 1;46(7):e70080. doi: 10.1002/hbm.70080 (PMC12044599; doi:10.1002/hbm.70080)
Supplement: Supplementary file 1 — Data S1: Supplementary Information. [file HBM-46-e70080-s001.docx]

**Supplementary Material**

1. Cross-correlation analysis among quality measures
2. Quality measures: range of values and inter-individual variability
3. FD-FDDVARS and FD-Q correlation analysis
4. Summary performance index: main formulas
5. Pairwise comparisons among the different denoising pipelines for each performance metric, for each metric category, and for the summary performance index.
   1. Noise-sensitive metrics
      1. FD-DVARS
      2. HF-FC content
   2. Network-sensitive metrics
      1. Modularity Q
      2. Time-based FCC
      3. Frequency-based FCC.
   3. Signal-sensitive metrics
      1. LF BOLD content
      2. tDOF loss
   4. Noise-sensitive, network-sensitive, and signal-sensitive metrics
   5. Summary performance index
6. Summary performance index: combination of quality measures with geometric mean
7. Summary performance index: noise-sensitive measures and positive measures
8. Supporting data and figures
9. Single-network time-based FCC
10. Simulation pilot study
11. **Cross-correlation between quality metrics**

The correlation among the various metrics was explored to study how the different metrics relate to each other and their collective impact on assessing denoising performance.

The cross correlation among the different performance metrics, in normalized values, was computed across subjects through the Pearson’s correlation coefficient. The same cross correlation was computed for all pipelines together and for each denoising pipeline separately. The results are visually shown in Fig. S1 and S2, respectively.

The results suggest that, within each category (i.e., noise-sensitive, network-sensitive, and signal-sensitive), the different measures are correlated positively each other. This is mainly true for the signal-sensitive category, even if it is more clearly shown only for pipelines including ICA-AROMA that have a variable number of tDOF. The correlation between the two noise-sensitive measures is positive but quite low, suggesting that indeed they quantify different aspects in artifact removal: FD-DVARS evaluates the residual motion artifacts effect, whereas HF-FC content quantifies the removal of fMRI artifacts in general, also including scanner-related artifacts and physiological noise. Within network-sensitive measures, Q and time-based FCC showed a strong positive correlation for each pipeline. This result is in accordance with the fact that both Q and FCC evaluate the segregation of whole-brain connectivity in different modules (Q), or more specifically in RSNs (FCC). Instead, frequency-based FCC presented a positive but lower correlation value with the other two, confirming its different nature for RSNs identification in the frequency domain.

Considering the between-category correlations, noise-sensitive measures (mainly HF-FC content) seemed to be inversely correlated with signal-sensitive ones across the different pipelines. Additionally, mainly for some pipelines, the correlation between network-sensitive measures and signal-sensitive measures presented negative values, confirming the appropriateness of including both signal-sensitive and network-sensitive categories, separately, in our summary performance index.


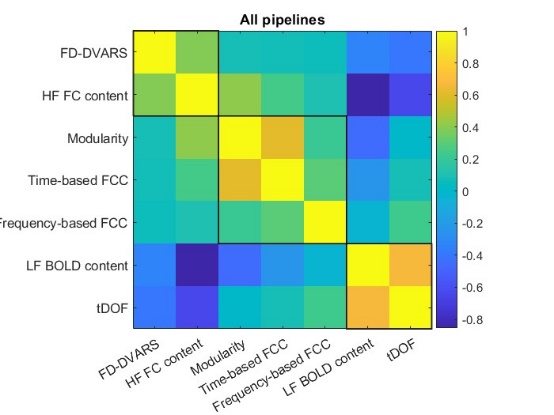


**Fig. S1. Cross-correlation among the various measures.** The correlation matrices representing the correlation among the different performance measures are reported in the figure. The black-line squares along the main diagonal group on noise-sensitive, network-sensitive, and signal-sensitive measures.


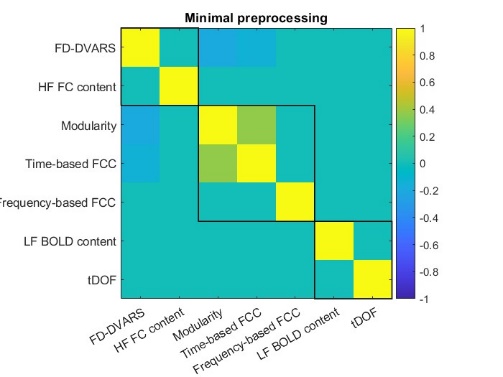


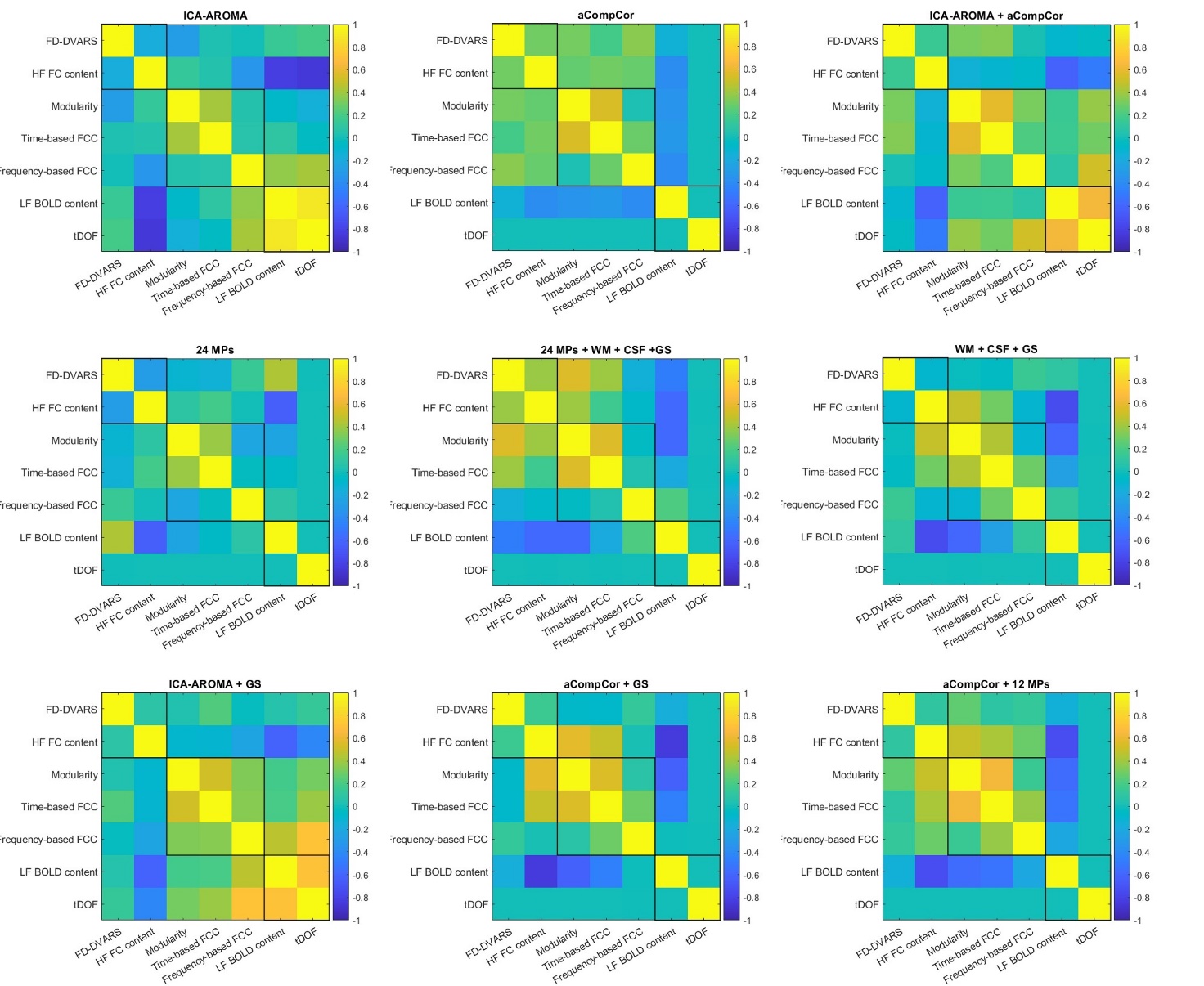


**Fig. S2. Cross-correlation among the various measures separately for each denoising pipeline.** The correlation matrices representing the correlation among the different performance measures are reported in the figure. The black-line squares along the main diagonal group on noise-sensitive, network-sensitive, and signal-sensitive measures.

1. **Quality measures: range of values and inter-individual variability**

**Range of values**

In this section, the ranges of expected values for the various quality metrics are presented, along with inverted values (for FD-DVARS and tDOF loss measures) and with the z-score normalized values.

**Noise-sensitive metrics**


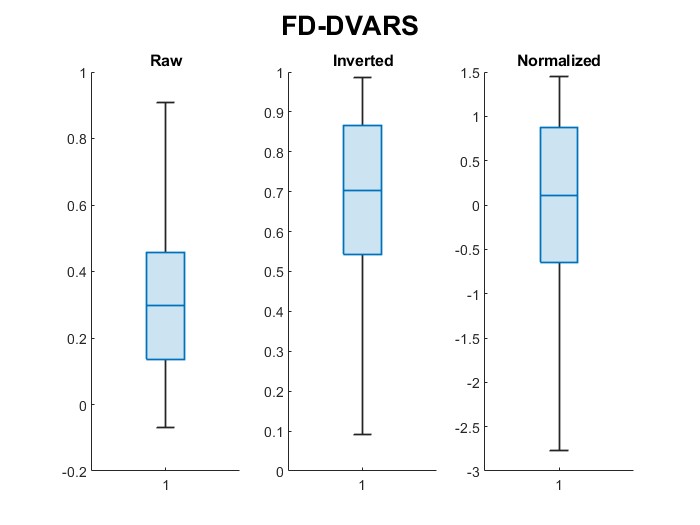

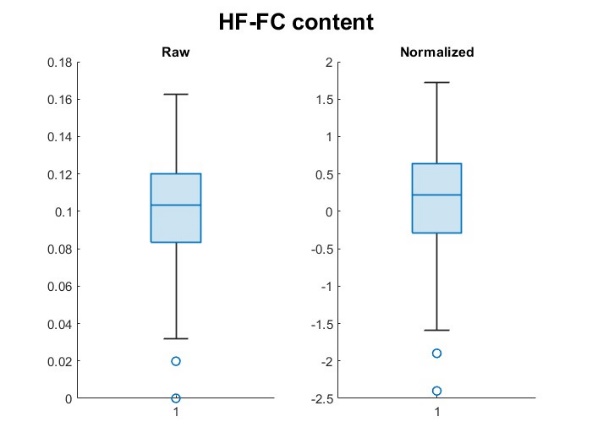


**Network-sensitive metrics**

**
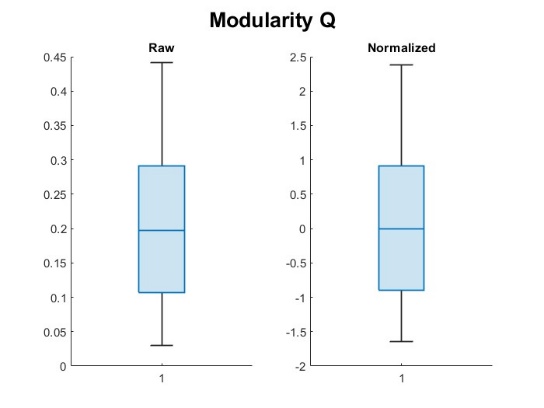

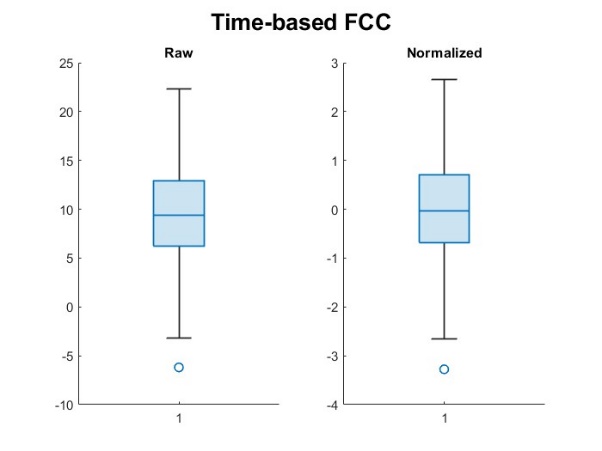

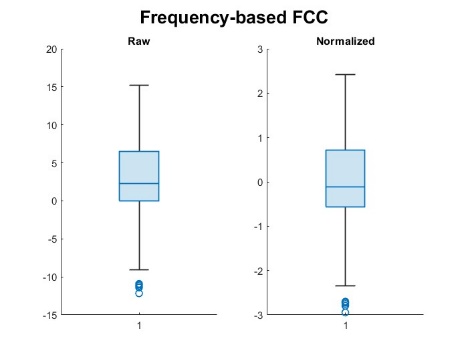
**

**Signal-sensitive metrics**


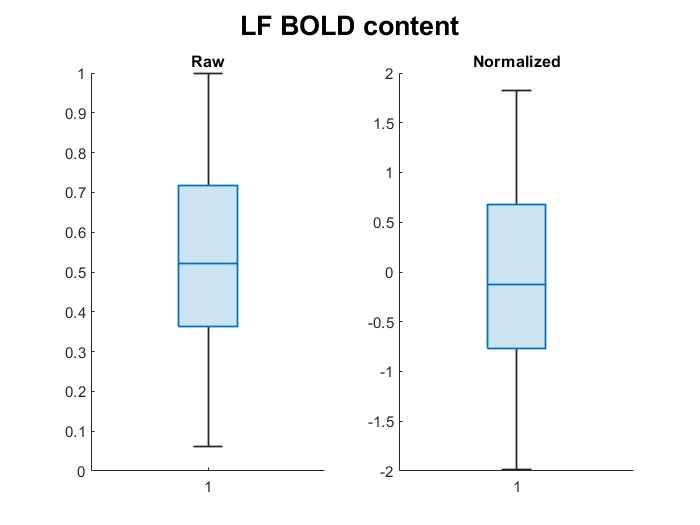

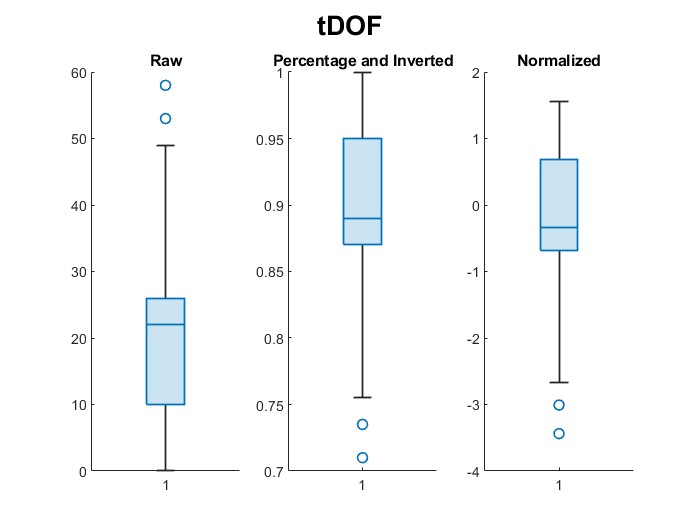


**Fig. S3. Range of values for the quality measures: noise-sensitive, network-sensitive, signal-sensitive categories.**

**Inter-individual variability**

To evaluate the effect of denoising on individual variability, a comparative analysis between denoised data and minimally pre-processed data was conducted, given the importance of preserving individual variability, as it plays a critical role in understanding cognitive and neurological processes.

We performed a comparative analysis of a set of metrics of interest before and after denoising. Specifically, considering that our dataset was acquired in resting-state condition, we compared key metrics in RSNs identification (modularity (Q) and FCC). We performed Levene’s test to compare statistically the variances before and after denoising. The null hypothesis of this test is that the data from the two conditions have the same variance. As shown in table T.S1, our results showed that the variance of the metrics was not significantly reduced after denoising. Levene’s test indicated no significant difference in variances (p > 0.005, 0.05 corrected for Bonferroni), suggesting that denoising does not substantially decrease the natural variability among participants. These analyses confirmed that while denoising enhances data quality, it does not seem to compromise the study of individual differences.

**Table T.S1. Results of Levene’s test for Q and FCC measures before and after denoising.**

|  | Denoising pipelines | | | | | | | | | |
| --- | --- | --- | --- | --- | --- | --- | --- | --- | --- | --- |
|  | **Baseline** | **ICA-AROMA** | **aCompCor** | **ICA-AROMA + aCompCor** | **24 MPs** | **24 MPs + WM + CSF + GS** | **WM + CSF + GS** | **ICA-AROMA + GS** | **aCompCor + GS** | **aCompCor + 12 MPs** |
| Q:  *p-value* | - | 0.98 | 0.63 | 0.69 | 0.36 | 0.62 | 0.11 | 0.033 | 0.0050 | 0.0086 |
| FCC:  *p-value* | - | 0.17 | 0.13 | 0.35 | 0.74 | 0.91 | 0.84 | 0.77 | 0.36 | 0.039 |

The p-values of Levene’s test comparing the variances of Q and FCC measures before and after denoising are reported in the table.

1. **Results of the correlation analysis between FD and FD-DVARS and between FD and Q**

**Correlation between FD and FD-DVARS**

**Table T.S2. FD-FDDVARS correlation results**

|  | Denoising pipelines | | | | | | | | | |
| --- | --- | --- | --- | --- | --- | --- | --- | --- | --- | --- |
|  | **Baseline** | **ICA-AROMA** | **aCompCor** | **ICA-AROMA + aCompCor** | **24 MPs** | **24 MPs + WM + CSF + GS** | **WM + CSF + GS** | **ICA-AROMA + GS** | **aCompCor + GS** | **aCompCor + 12 MPs** |
| *Correlation r* | 0.280 | 0.347 | -0.036 | 0.031 | 0.304 | 0.165 | -0.078 | 0.271 | -0.190 | 0.302 |
| *p-value* | 0.042 | 0.011 | 0.801 | 0.825 | 0.027 | 0.237 | 0.570 | 0.050 | 0.174 | 0.029 |

The results of the correlation analysis between FD and FD-DVARS are reported in the table.

**Correlation between FD and Q**

**Table T.S3. FD-Q correlation results**

|  | Denoising pipelines | | | | | | | | | |
| --- | --- | --- | --- | --- | --- | --- | --- | --- | --- | --- |
|  | **Baseline** | **ICA-AROMA** | **aCompCor** | **ICA-AROMA + aCompCor** | **24 MPs** | **24 MPs + WM + CSF + GS** | **WM + CSF + GS** | **ICA-AROMA + GS** | **aCompCor + GS** | **aCompCor + 12 MPs** |
| *Correlation r* | -0.0078 | 0.258 | 0.199 | 0.049 | 0.214 | -0.299 | -0.208 | -0.175 | -0.132 | -0.259 |
| *p-value* | 0.956 | 0.062 | 0.153 | 0.730 | 0.123 | 0.029 | 0.134 | 0.209 | 0.346 | 0.062 |

The results of the correlation analysis between FD and Q are reported in the table.

1. **Summary performance index: main formulas**

Z-score normalization:

$$Metric normalized= \frac{Metric value-mean(metric)}{standard deviation(metric)}$$

Mean values of noise-, network-, signal-sensitive measures:

$$Mean noise = \frac{FDDVARS+HF FC content}{2}$$

$$Mean network = \frac{Q+time based FCC+frequency based FCC}{3}$$

$$Mean signal = \frac{LF BOLD content+tDOF loss}{2}$$

Summary performance index calculation:

$$Summary performance index= \frac{Mean noise+Mean network+Mean signal}{3}$$

1. **Pairwise comparisons among the different denoising pipelines**

**Pairwise comparisons among the different denoising pipelines for each performance metric**

**Noise-sensitive metrics**

- **FD-DVARS**


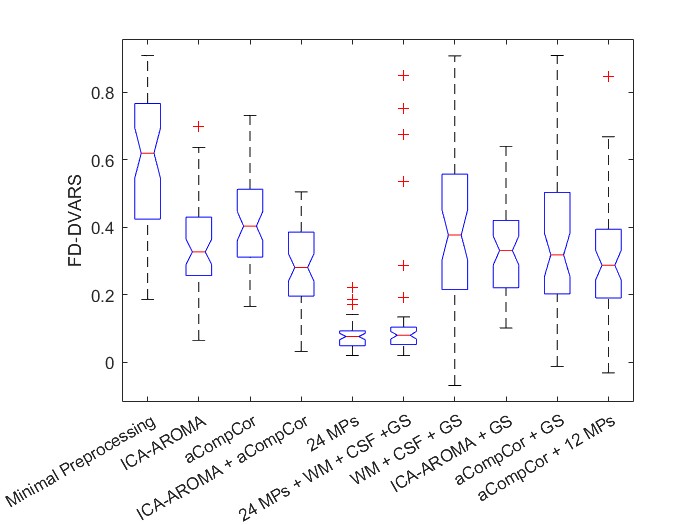

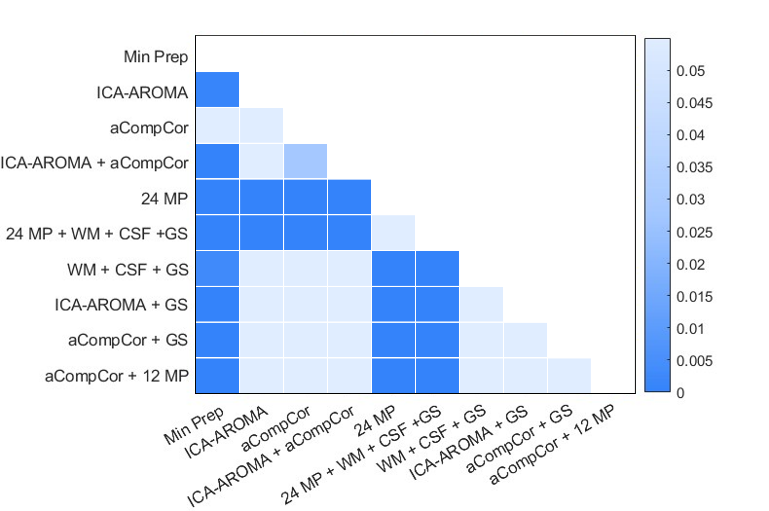


(a) (b)

**Fig. S4**. **FD-DVARS**. a) KW test boxplots. b) Pairwise comparisons among the different denoising pipelines. The pairwise p-values are reported as shown in the colorbar.

- **HF-FC content**


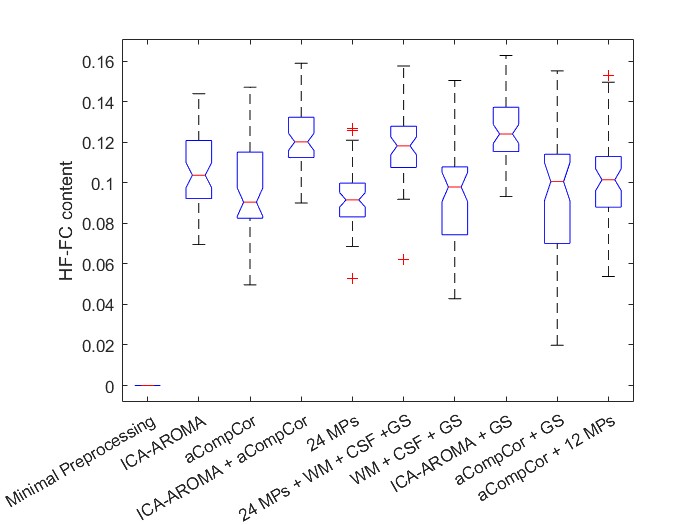

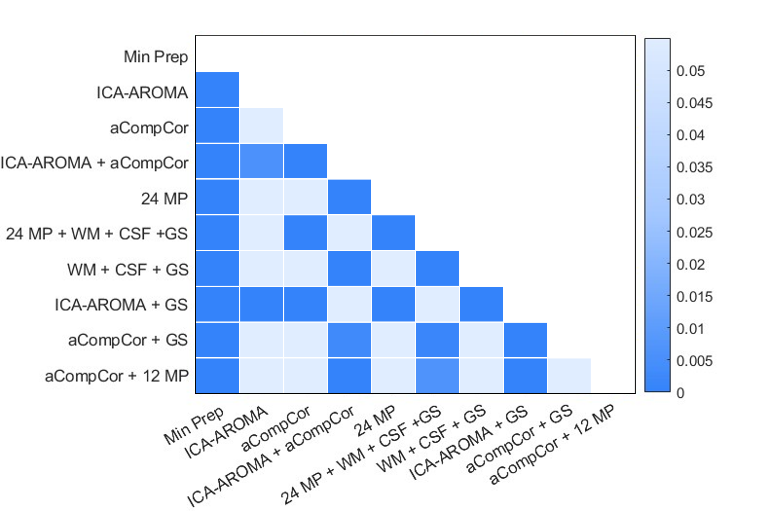


(a) (b)

**Fig. S5**. **HF-FC content**. a) KW test boxplots. b) Pairwise comparisons among the different denoising pipelines. The pairwise p-values are reported as shown in the colorbar.

**Network-sensitive metrics**

- **Modularity**


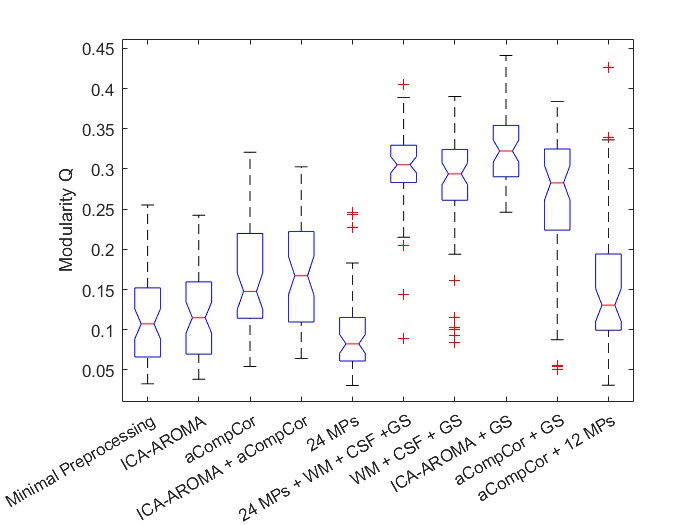

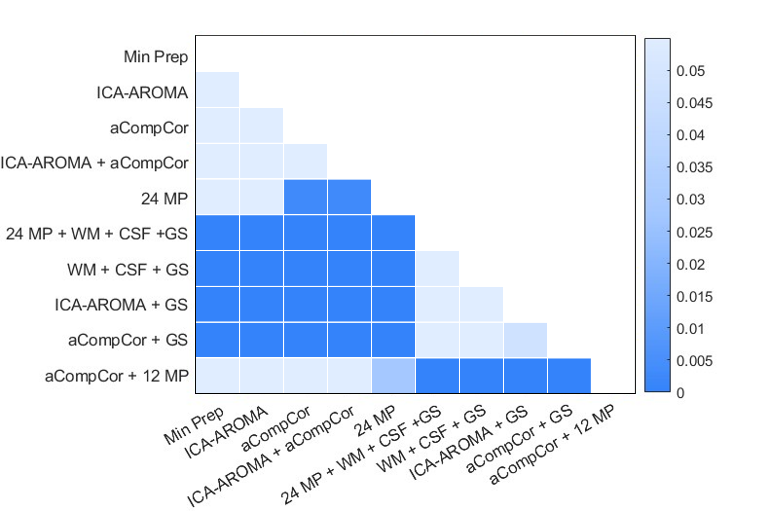


(a) (b)

**Fig. S6**. **Modularity**. a) KW test boxplots. b) Pairwise comparisons among the different denoising pipelines. The pairwise p-values are reported as shown in the colorbar.

- **Time-based FCC**


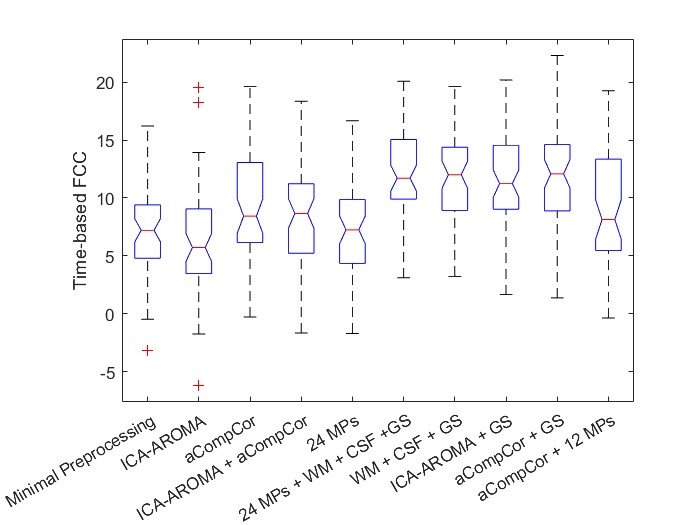

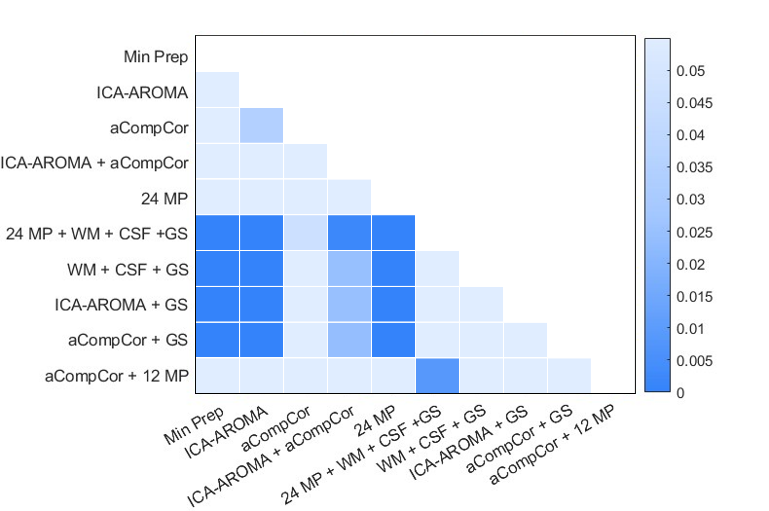


(a) (b)

**Fig. S7**. **Time-based FCC**. a) KW test boxplots. b) Pairwise comparisons among the different denoising pipelines. The pairwise p-values are reported as shown in the colorbar.

- **Frequency-based FCC**


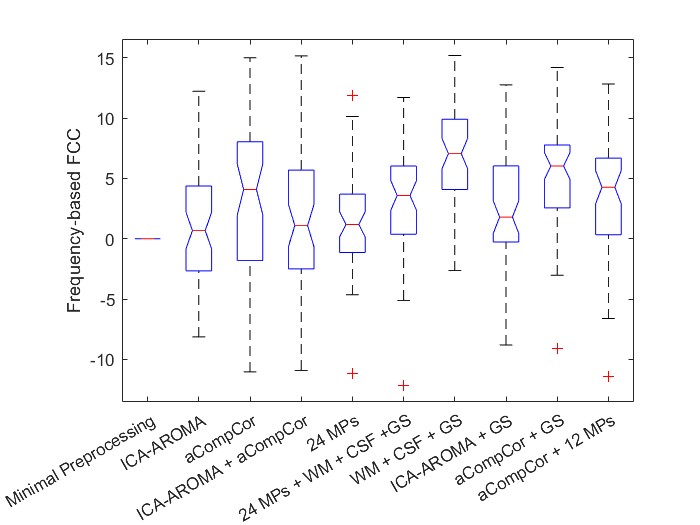

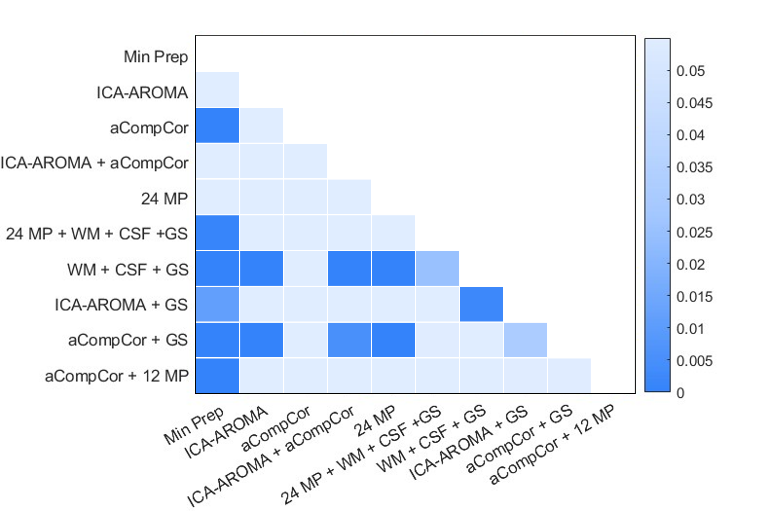


(a) (b)

**Fig. S8**. **Frequency-based FCC**. a) KW test boxplots. b) Pairwise comparisons among the different denoising pipelines. The pairwise p-values are reported as shown in the colorbar.

**Signal-sensitive metrics**

- **LF BOLD content**


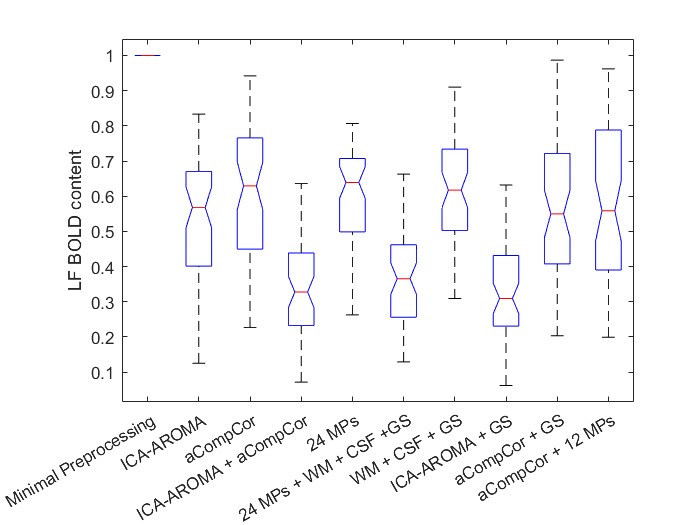

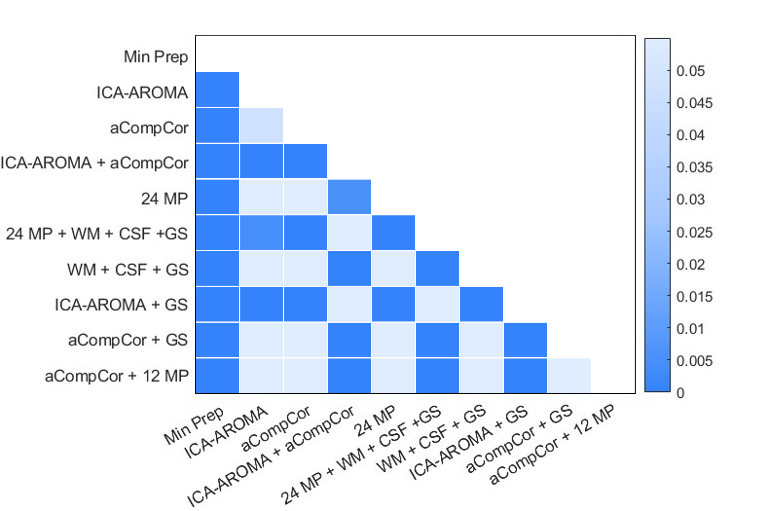


(a) (b)

**Fig. S9**. **LF BOLD content.** a) KW test boxplots. b) Pairwise comparisons among the different denoising pipelines. The pairwise p-values are reported as shown in the colorbar.

- **tDOF loss**


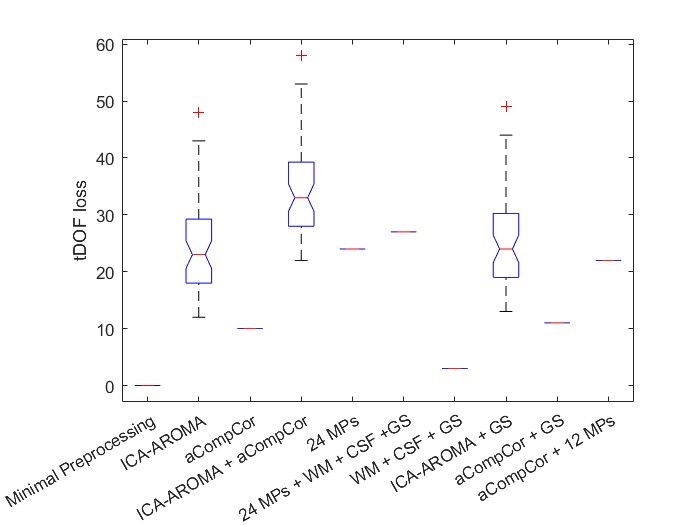

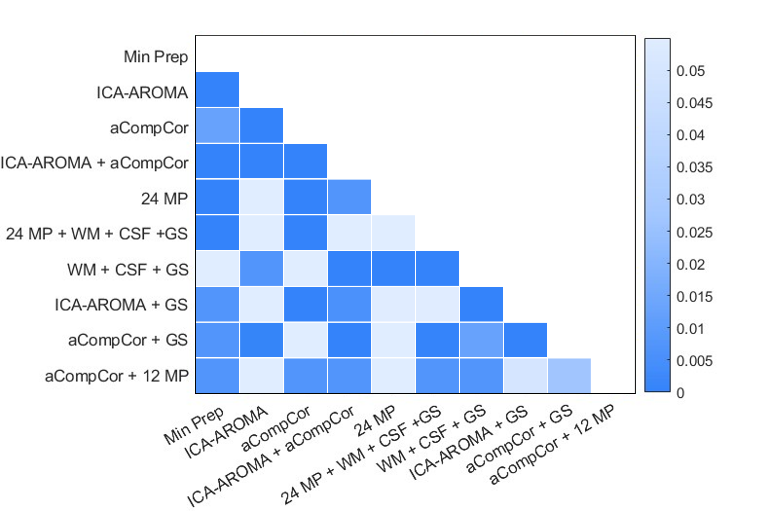


(a) (b)

**Fig. S10**. **tDOF loss.** a) KW test boxplots. b) Pairwise comparisons among the different denoising pipelines. The pairwise p-values are reported as shown in the colorbar.

**Pairwise comparisons among the different denoising pipelines for each metric category**

- **Noise-sensitive measures**


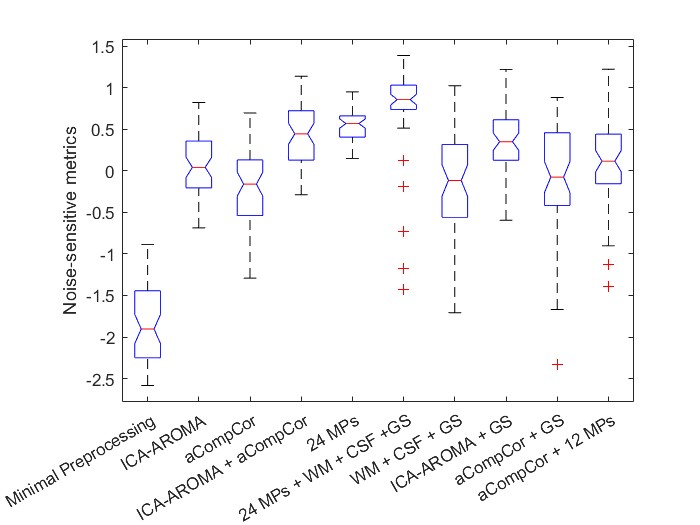

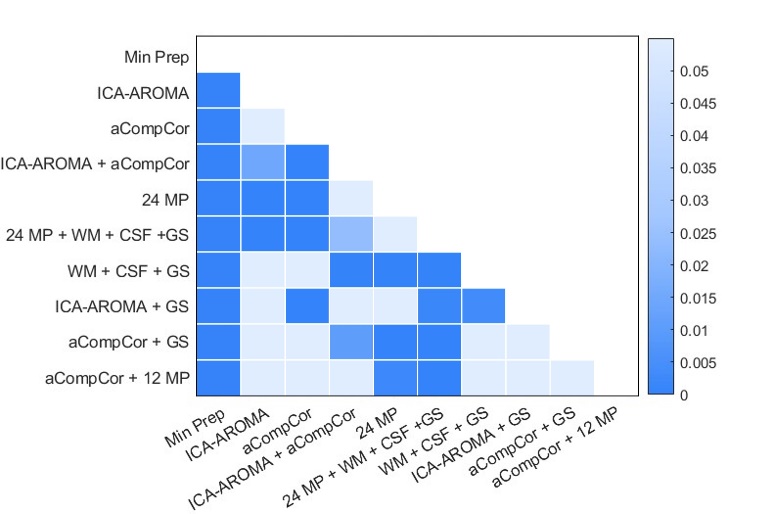


**Fig. S11**. **Noise-sensitive measures.** a) KW test boxplots. b) Pairwise comparisons among the different denoising pipelines. The pairwise p-values are reported as shown in the colorbar.

- **Network-sensitive measures**

**
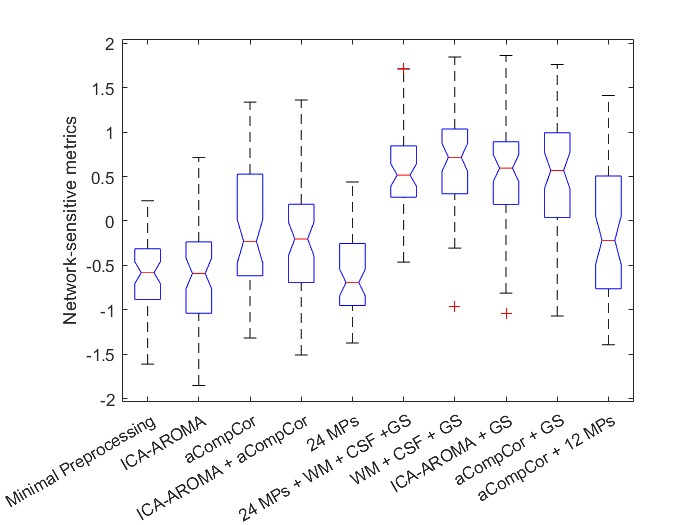

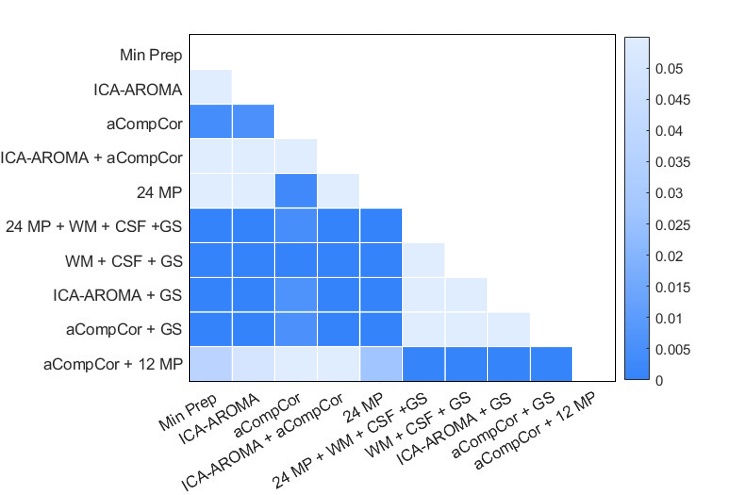
**

**Fig. S12**. **Network-sensitive measures.** a) KW test boxplots. b) Pairwise comparisons among the different denoising pipelines. The pairwise p-values are reported as shown in the colorbar.

- **Signal-sensitive measures**


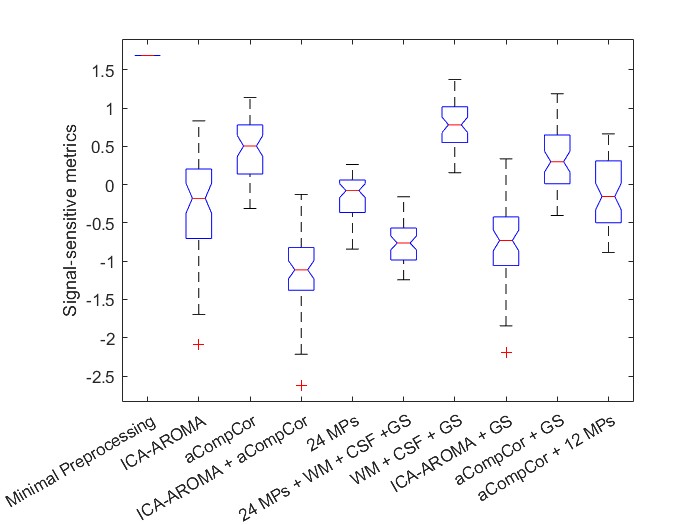

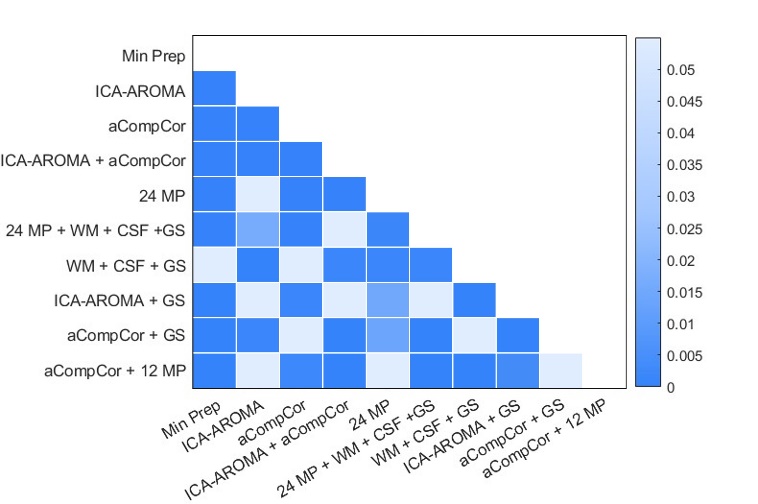


**Fig. S13**. **Signal-sensitive measures.** a) KW test boxplots. b) Pairwise comparisons among the different denoising pipelines. The pairwise p-values are reported as shown in the colorbar.

**Pairwise comparisons among the different denoising pipelines for the summary performance index**

- **Summary performance index**


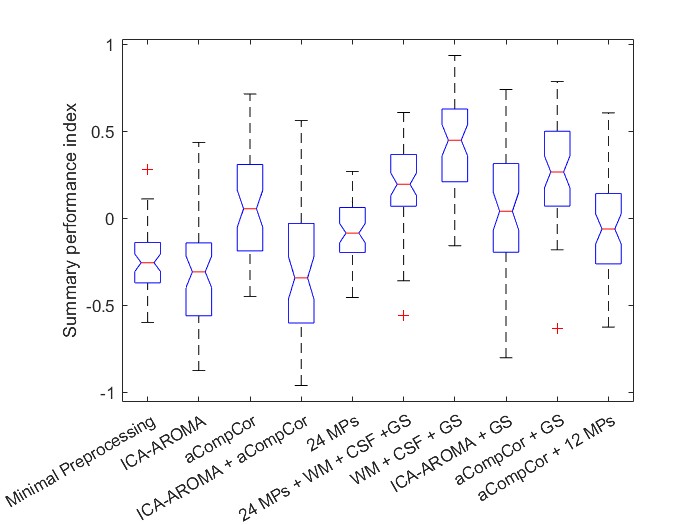

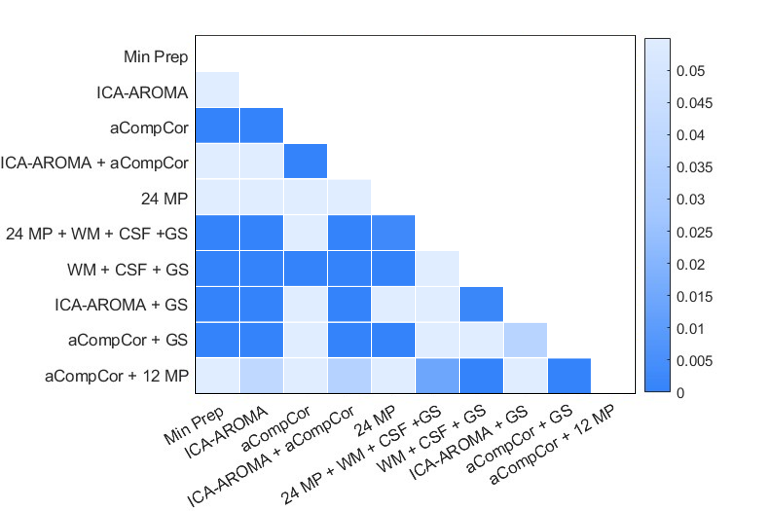


(a) (b)

**Fig. S14**. **Summary performance index.** a) KW test boxplots. b) Pairwise comparisons among the different denoising pipelines. The pairwise p-values are reported as shown in the colorbar.

1. **Summary performance index: combination of quality measures with geometric mean**

Noise-sensitive, network-sensitive, and signal-sensitive measures were aggregated to achieve a composite performance index that could summarize the results of the previous metrics.

As described in the main text, the combination of the different measures was performed by computing the arithmetic mean of the single measures. Considering that the arithmetic mean could allow for compensation between metrics, we wanted to test if the application of a different approach would have made a difference in the results. Therefore, in this case, we combined the different measures by computing the geometric mean, which should return a high value only if all the inputs are high.

**Methods**

As reported in the main manuscript, FD-DVARS and tDOF loss were normalized to 1-abs(x), where x is the score of each metric, so that, similarly to the other measures, a high positive score is assigned to good quality data. Subsequently, each metric was normalized with a min-max normalization (i.e., subtracting the minimum value and dividing by the difference between the maximum and minimum values). Differently from our first approach, in which a z-score normalization was applied, in this case we applied a normalization that report the values between the 0 and 1 because the geometric mean works only with positive values.

The normalized metrics were then used to compute a summary quality index as follows. The geometric mean values of noise-sensitive, network-sensitive, and signal-sensitive measures were separately computed. Then, the summary performance index was computed as the geometric mean of the above three mean values.

The same statistical analysis that was described for the first version of the summary performance index was applied to this measure.

**Results**

The KW comparison based on the summary index reported significant differences among pipelines (p<0.05, Bonferroni corrected). The median, and the first and third quartiles and the corresponding KW statistics are listed in Table T.S4. The boxplots of the summary performance index for all denoising pipelines are reported in Fig. S15. The post-hoc multiple comparisons showed that the denoising method with WM + CSF + GS regression outperformed the other denoising techniques. This pipeline performed better than the baseline preprocessing, pipelines including ICA-AROMA, 24 MPs, aCompCor and aCompCor + 12 MPs, which were associated with significantly lower values of the summary performance index, and it resulted comparable to 24 MPs + WM + CSF + GS and to aCompCor + GS.

**Table T.S4. Results of the summary performance index for each denoising pipeline.**

| Performance metrics | Denoising pipelines (1^st^ quartile, median, 3^rd^ quartile) | | | | | | | | | | KW test  (*p-value*) |
| --- | --- | --- | --- | --- | --- | --- | --- | --- | --- | --- | --- |
|  | **Baseline** | **ICA-AROMA** | **aCompCor** | **ICA-AROMA + aCompCor** | **24 MPs** | **24 MPs + WM + CSF + GS** | **WM + CSF + GS** | **ICA-AROMA + GS** | **aCompCor + GS** | **aCompCor + 12 MPs** |  |
| Summary performance index | 0  0  0 | 0.399  **0.466**  0.526 | 0.484  **0.545**  0.609 | 0.391  **0.451**  0.528 | 0.457  **0.497**  0.061 | 0.531  **0.565**  0.617 | 0.578  **0.627**  0.685 | 0.486  **0.543**  0.607 | 0.539  **0.599**  0.644 | 0.480  **0.532**  0.565 | 2.987e-50 |

Results of the summary performance index (with geometric mean) for each denoising pipeline after min-max normalization. The first quartile, the median and the third quartile and the corresponding KW statistics are listed.

**
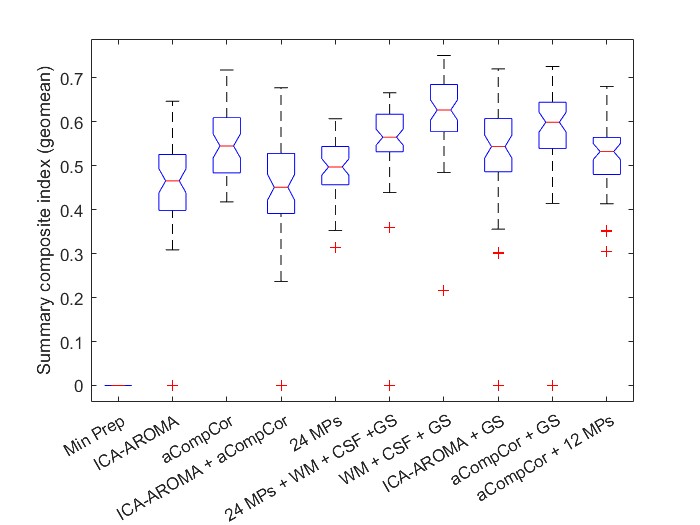

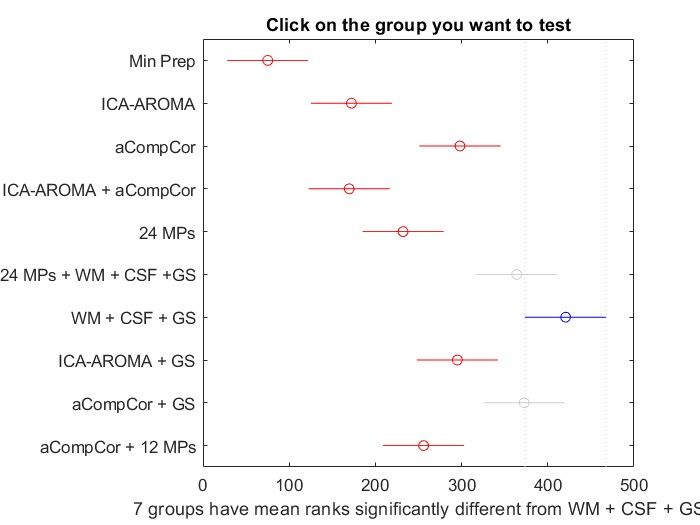
**

(a) (b)

**Fig. S15**. **Summary performance index (geometric mean).** a) KW test boxplots. b) Pairwise comparisons among the different denoising pipelines.

**Discussion**

The calculation of a summary composite index with the geometric mean showed results that in line with the results obtained with the application of the arithmetic mean, reported in the main text. The results computed with the geometric mean are supportive of a composite index that reflects consistent performance across all metrics; and the comparison between the results obtained with the two different approaches suggest a robust evaluation of the methods we benchmarked.

Given the similarities, the discussion of the comparison among the different denoising pipelines can be considered similar to the one described in the main text.

**
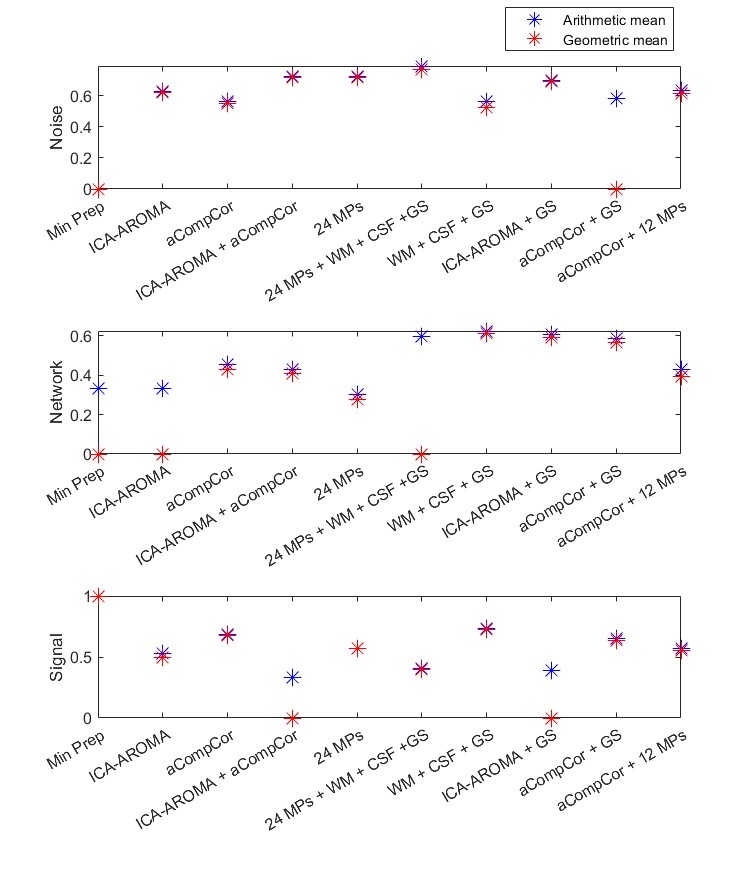
**

**Fig. S16**. **Mean of noise-sensitive, network-sensitive, and signal-sensitive measures with arithmetic and geometric means.**

1. **Summary performance index: noise-sensitive and positive measures**

Signal- and network-sensitive measures, both categorized as “positive” quality control measures, may overlap in their effects. Consequently, including separate values for these measures in the summary performance index could introduce a bias that favors signal preservation over artifact removal.

Therefore, a new summary composite index was thought to consider these aspects and provide a different point of view of quality control measures combination.

**Methods**

As detailed in the main text, FD-DVARS and tDOF loss measures were inverted by computing 1-abs(x), where x is the raw metric score, ensuring that higher scores correspond to better data quality, consistent with the other metrics. Next, all metrics were normalized using z-score normalization. The normalized metrics were then used to compute a summary quality index as follows.

The mean value of noise-sensitive metrics was computed. The mean values of Q, time-based FCC, frequency-based FCC, LF BOLD content, and tDOF were computed. Finally, the summary performance index was computed as the mean value of the noise-sensitive and “positive” mean values.

**Results**

The KW comparison based on the summary index reported significant differences among pipelines (p<0.05, Bonferroni corrected). The median, and the first and third quartiles and the corresponding KW statistics are listed in Table T.S5. The boxplots of the summary performance index for all denoising pipelines are reported in Fig. S17.

The post-hoc multiple comparisons showed that the denoising method 24 MPs + WM + CSF + GS outperformed the other denoising techniques. This pipeline performed better than the baseline preprocessing, ICA-AROMA, aCompCor, ICA-AROMA + aCompCor, 24 MPs, and aCompCor + 12 MPs, which were associated with significantly lower values of the summary performance index, and it resulted non significantly different from WM + CSF + GS, ICA-AROMA + GS, and aCompCor + GS.

**Table T.S5. Results of the summary performance index for each denoising pipeline.**

| Performance metrics | Denoising pipelines (1^st^ quartile, median, 3^rd^ quartile) | | | | | | | | | | KW test  (*p-value*) |
| --- | --- | --- | --- | --- | --- | --- | --- | --- | --- | --- | --- |
|  | **Baseline** | **ICA-AROMA** | **aCompCor** | **ICA-AROMA + aCompCor** | **24 MPs** | **24 MPs + WM + CSF + GS** | **WM + CSF + GS** | **ICA-AROMA + GS** | **aCompCor + GS** | **aCompCor + 12 MPs** |  |
| Summary performance index | -0.941  -0.782  -0.623 | -0.434  **-0.234**  -0.049 | -0.325  **-0.058**  **0.267** | -0.341  -0.105  0.166 | -0.068  **0.041**  0.194 | 0.332  **0.448**  0.554 | 0.045  **0.269**  0.549 | -0.050  **0.107**  0.435 | -0.123  **0.234**  0.488 | -0.235  **-0.047**  0.217 | 7.691e-43 |

Results of the noise-signal summary performance index for each denoising pipeline after z-score normalization. The first quartile, the median and the third quartile and the corresponding KW statistics are listed.


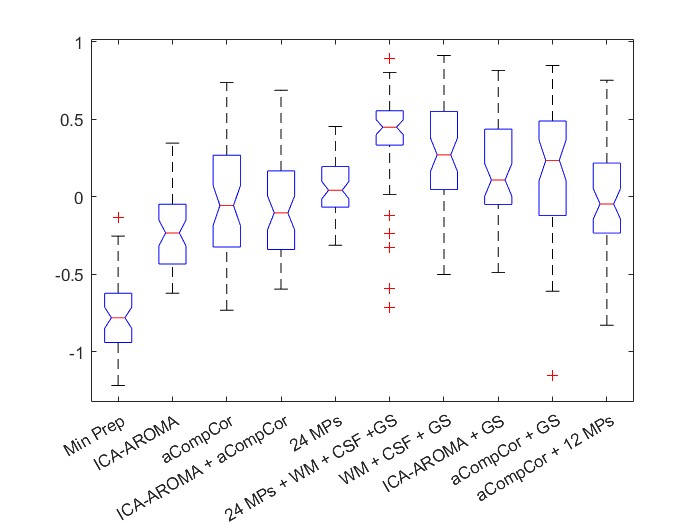

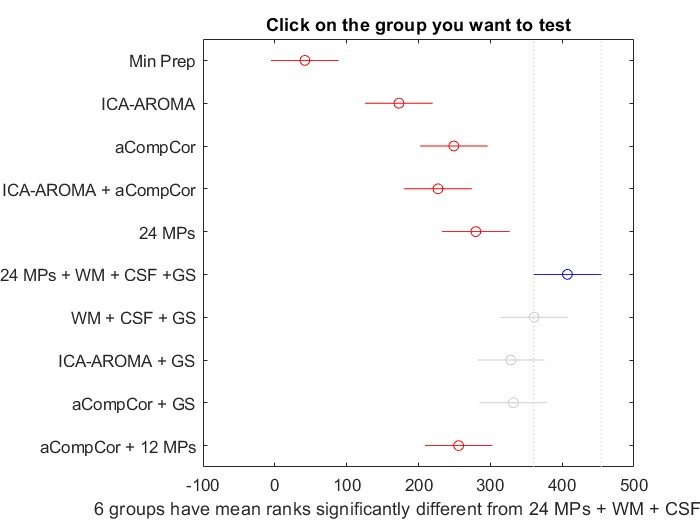


(a) (b)

**Fig. S17**. **Summary performance index (geometric mean).** a) KW test boxplots. b) Pairwise comparisons among the different denoising pipelines.

**Discussion**

The results of the noise-signal summary performance index indicated that the pipeline 24 MPs + WM + CSF + GS achieved the highest performance score, consistent with the other pipelines including GS (i.e., WM + CSF + GS, ICA-AROMA + GS, and aCompCor + GS).

These findings closely aligned with those obtained using the first version of the summary composite index, which is described in the main text, which also favored WM + CSF + GS, 24 MPs + WM + CSF + GS, and aCompCor + GS. The only difference lies in the ICA-AROMA + GS pipeline, that including ICA-AROMA noise-components regression resulted more aggressive on the signal preservation. As a result, this pipeline is less signal-friendly according to the main summary composite index.

Given the similarities, the discussion comparing the various denoising pipelines remains largely consistent with the one presented in the main text.

The cross-correlation analysis of the various quality control measures revealed that the three types of performance metrics – noise-sensitive, network-sensitive, and signal-sensitive - appeared to cover distinct characteristics in fMRI denoising, as no significant positive between-category correlations were observed. Given these assumptions, in the main text, the version of the summary index including noise-, network-, and signal-sensitive measures separately has been maintained, as it provides a more comprehensive assessment of the fMRI denoising methods.

However, considering that the selection of processing steps should ideally depend on the dataset and research objective, we also presented a more balanced noise-signal composite index, as a proposed alternative for situations where less conservative denoising pipelines are preferred.

1. **Supporting data**

In the present section, supporting data for our denoising methods comparison are presented for exemplar low-motion and high-motion subjects. The present figures include: grayplots, FD-DVARS plots, correlation matrices, wavelet coherence spectra, and power spectral densities.

**Low motion subject (mean FD=0.083)**

**Grayplot**

**
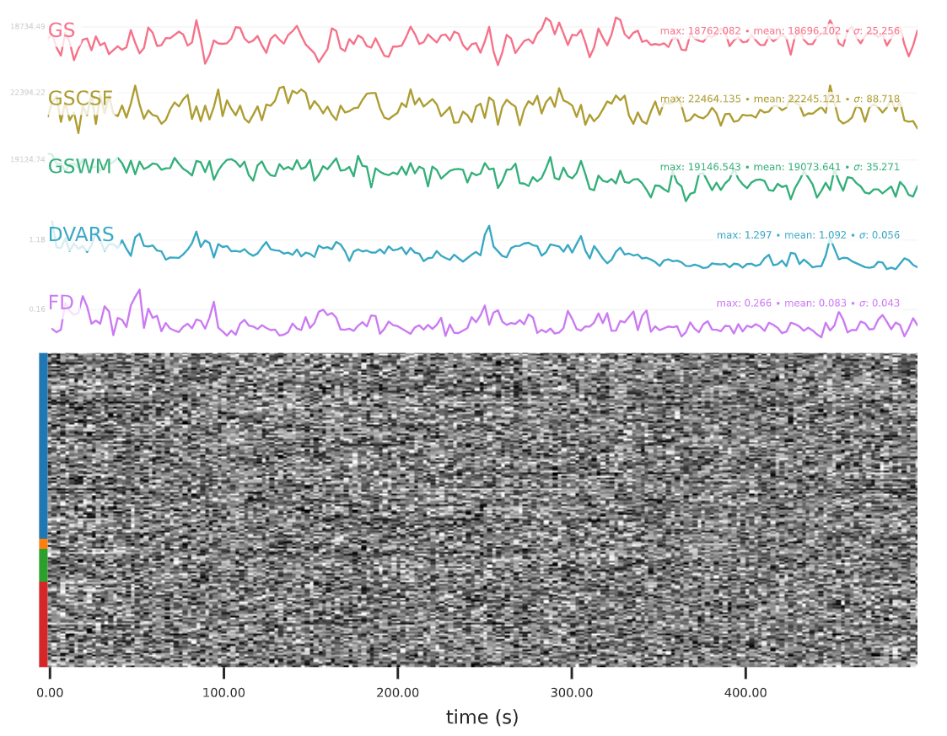
**

**Fig. S18. Grayplot for a low-motion subject.** A carpet plot is a two-dimensional plot of time series within a scan, with time on the x-axis and voxels on the y-axis. Voxels are grouped into cortical gray matter (blue), subcortical gray matter (orange), cerebellum (green), and white matter and cerebrospinal fluid (red). Above the carpet plot are time courses of the magnitude of FD, GS, global signal in CSF (GSCSF), global signal in WM (GSWM), and DVARS.

**FD-DVARS**

**
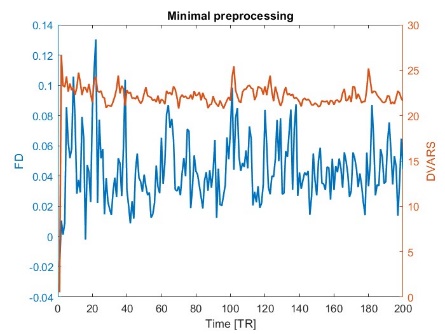
**

**
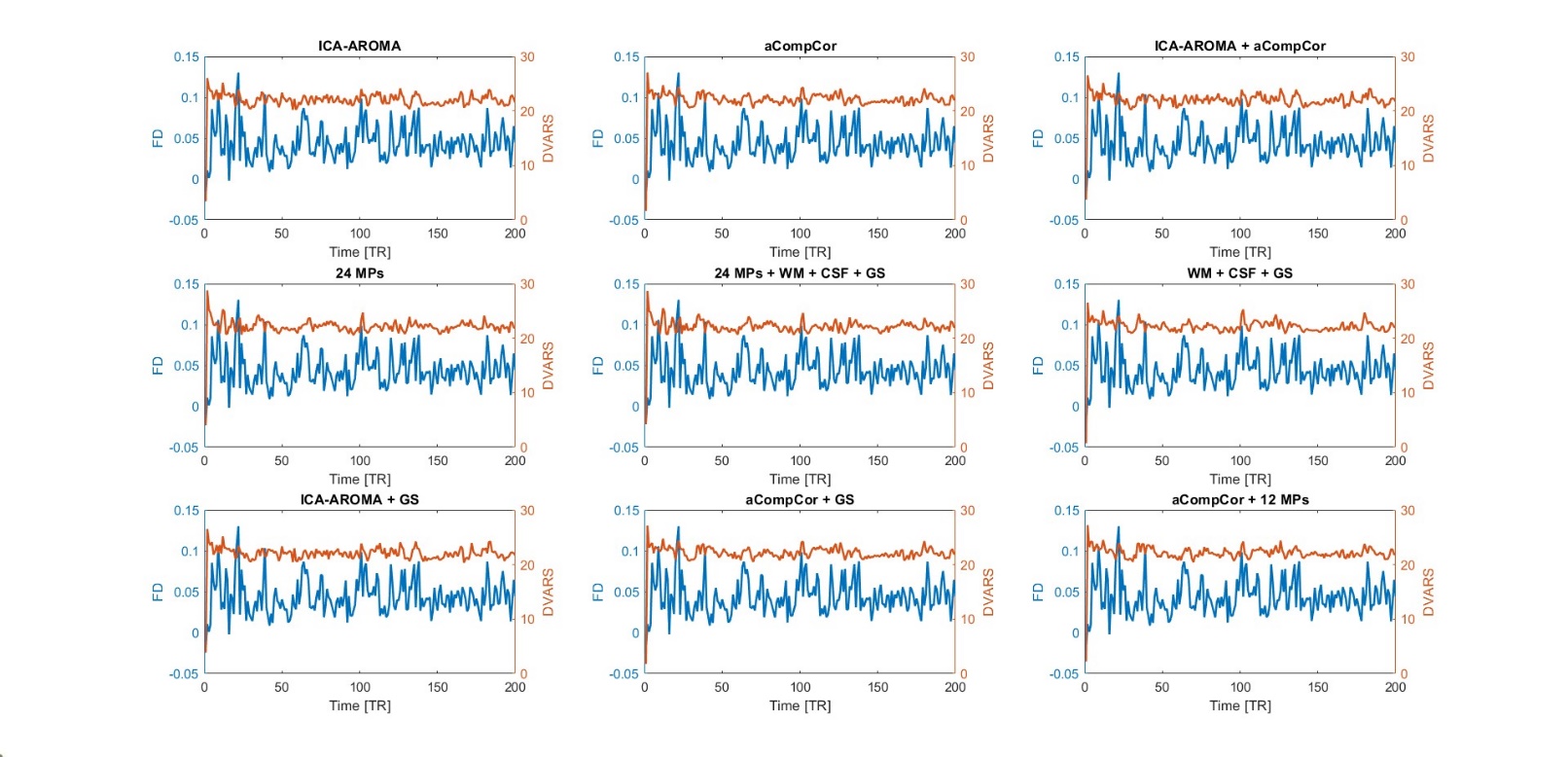
**

**Fig. S19. FD-DVARS plots for a low-motion subject.** FD and DVARS time series are shown for the minimal preprocessing and for each denoising pipeline.

**FC matrices**

**
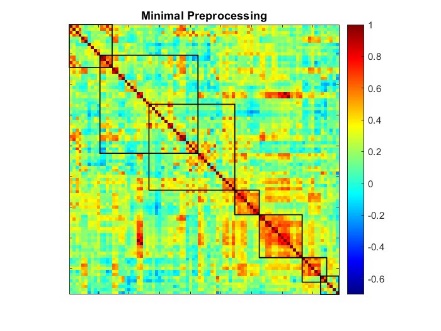

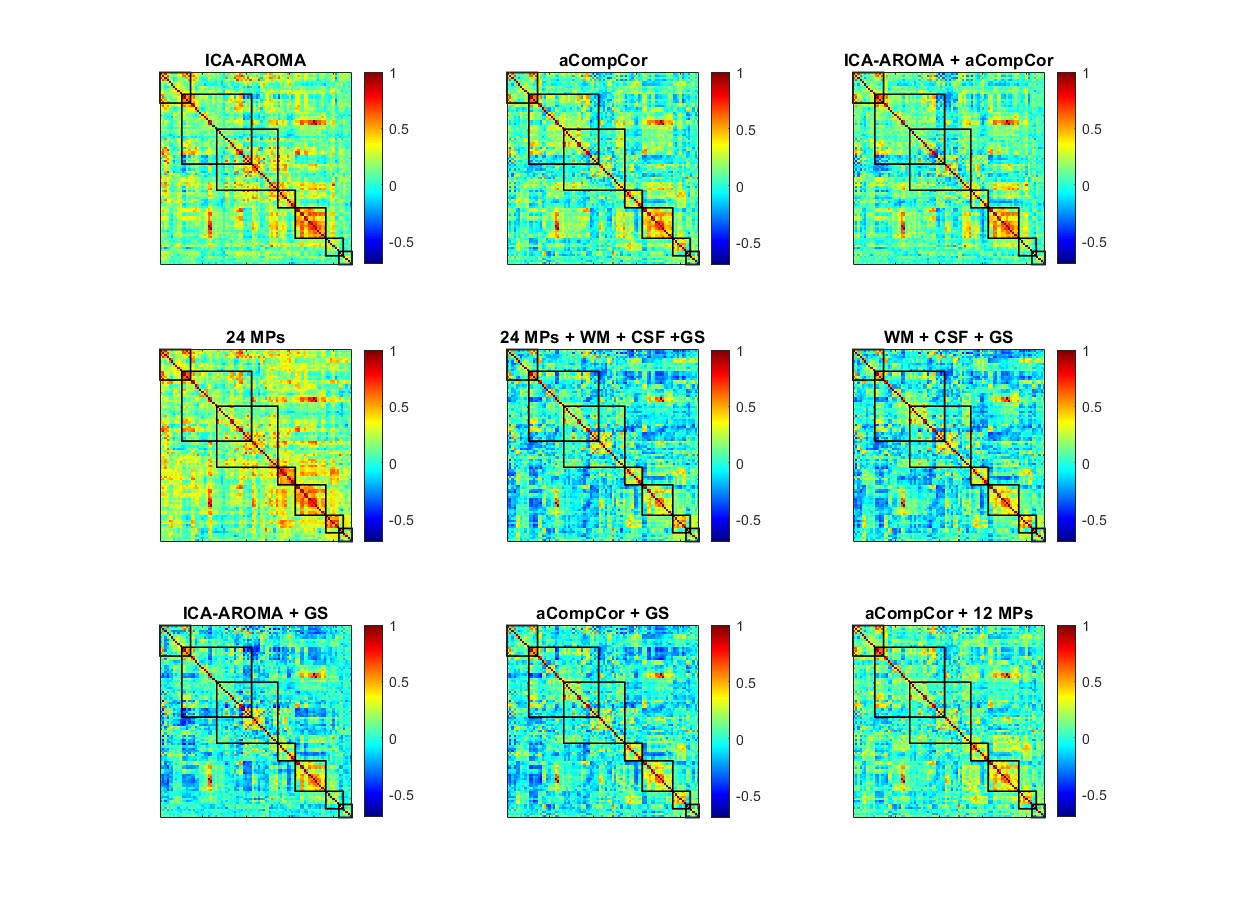
**

**Fig. S20. FC matrices for a low-motion subject.** Pairwise correlation matrices (Peason’s correlation coefficient), with ROIs reorganized in RSNs, are shown for the minimal preprocessing and for each denoising pipeline.

**Wavelet coherence spectra**

**
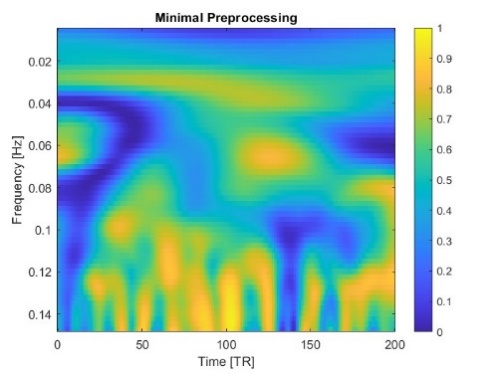
**

**
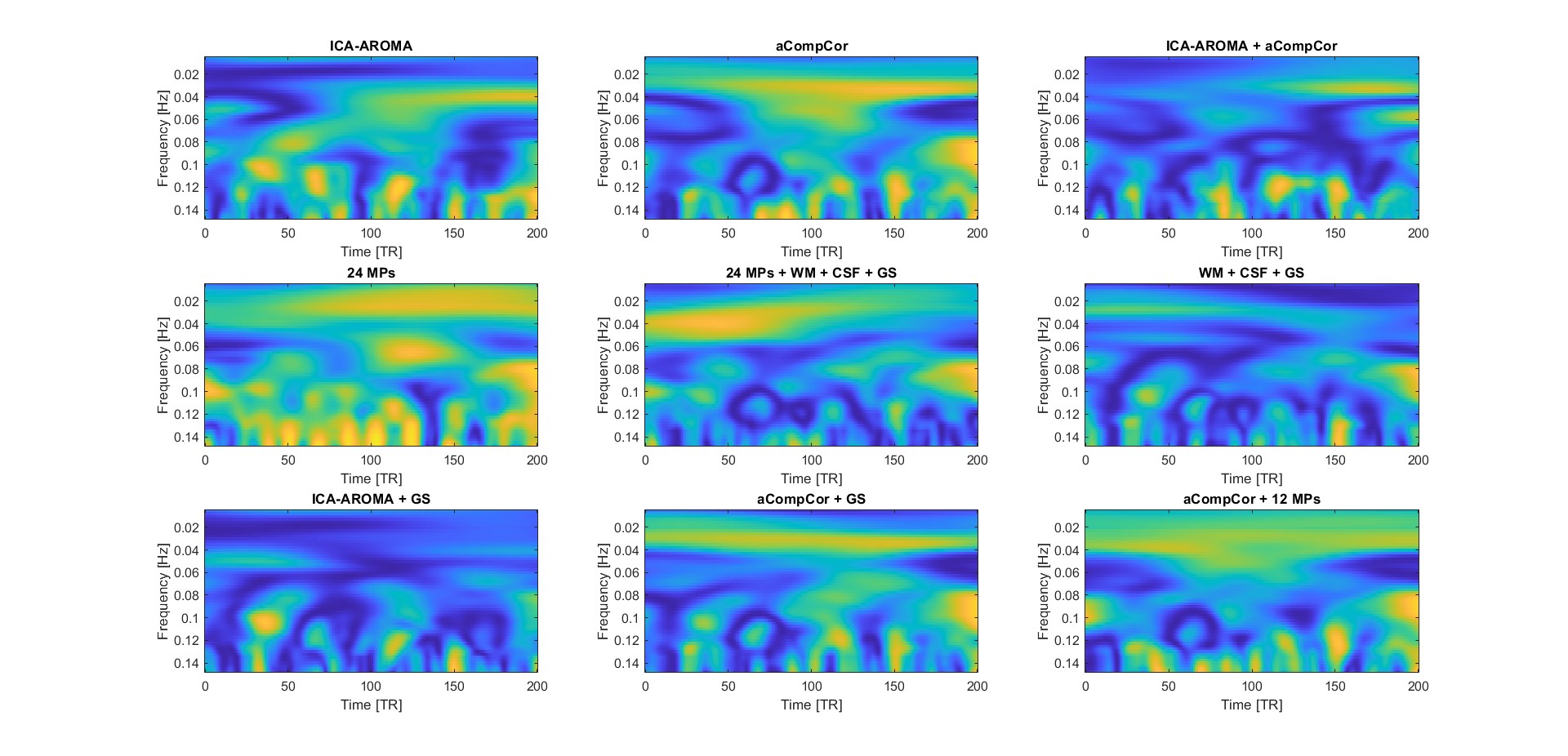
**

**Fig. S21. Wavelet coherence spectra for a low-motion subject.** Wavelet coherence spectra estimated from the BOLD signals of an exemplar pair of ROIs are shown for the minimal preprocessing and for each denoising pipeline.

**Power spectral densities**

**
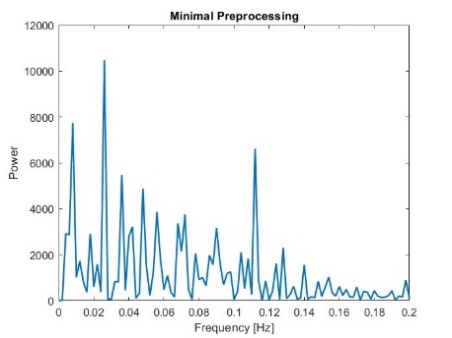
**

**
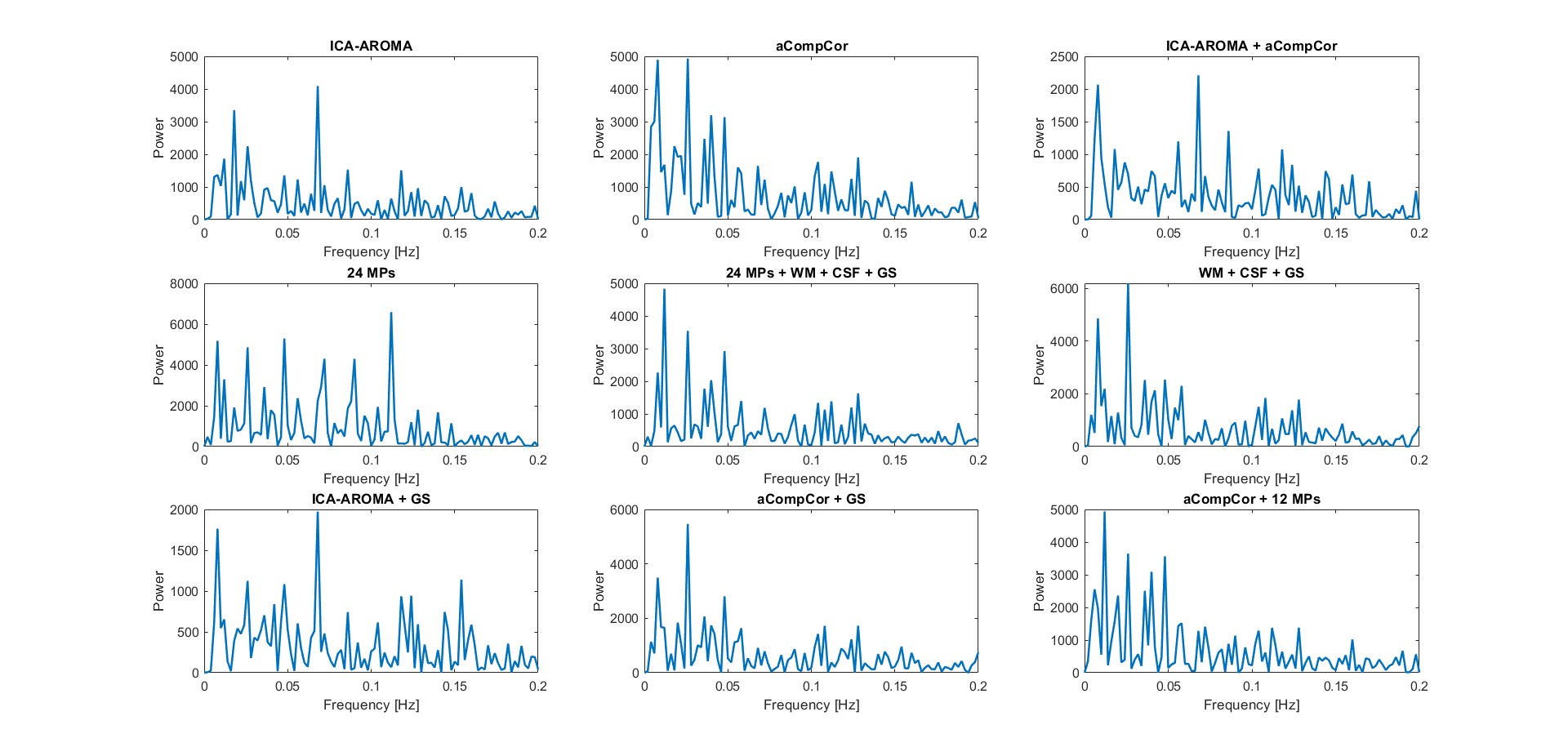
**

**Fig. S22. Power spectra for a low-motion subject.** Power spectral densities estimated from the BOLD signal of an exemplar ROI are shown for the minimal preprocessing and for each denoising pipeline.

**High motion subject (mean FD = 0.138)**

**Grayplot**


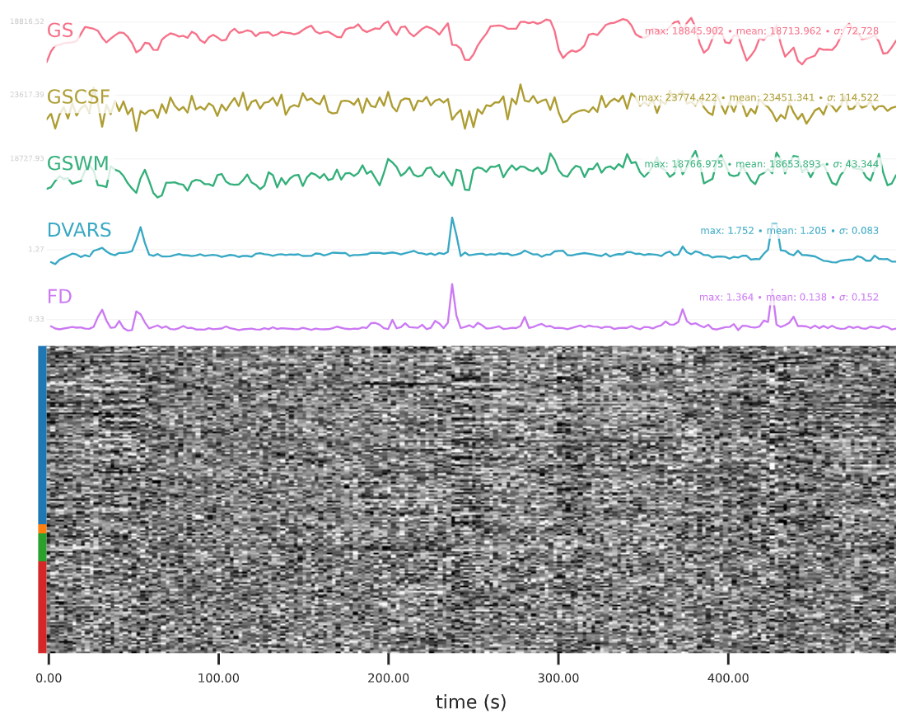


**Fig. S23. Grayplot for a high-motion subject.** A carpet plot is a two-dimensional plot of time series within a scan, with time on the x-axis and voxels on the y-axis. Voxels are grouped into cortical gray matter (blue), subcortical gray matter (orange), cerebellum (green), and white matter and cerebrospinal fluid (red). Above the carpet plot are time courses of the magnitude of FD, GS, global signal in CSF (GSCSF), global signal in WM (GSWM), and DVARS.

**FD-DVARS**


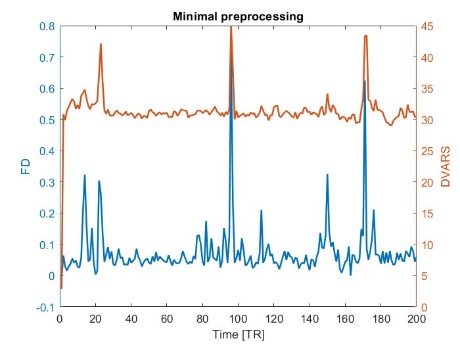


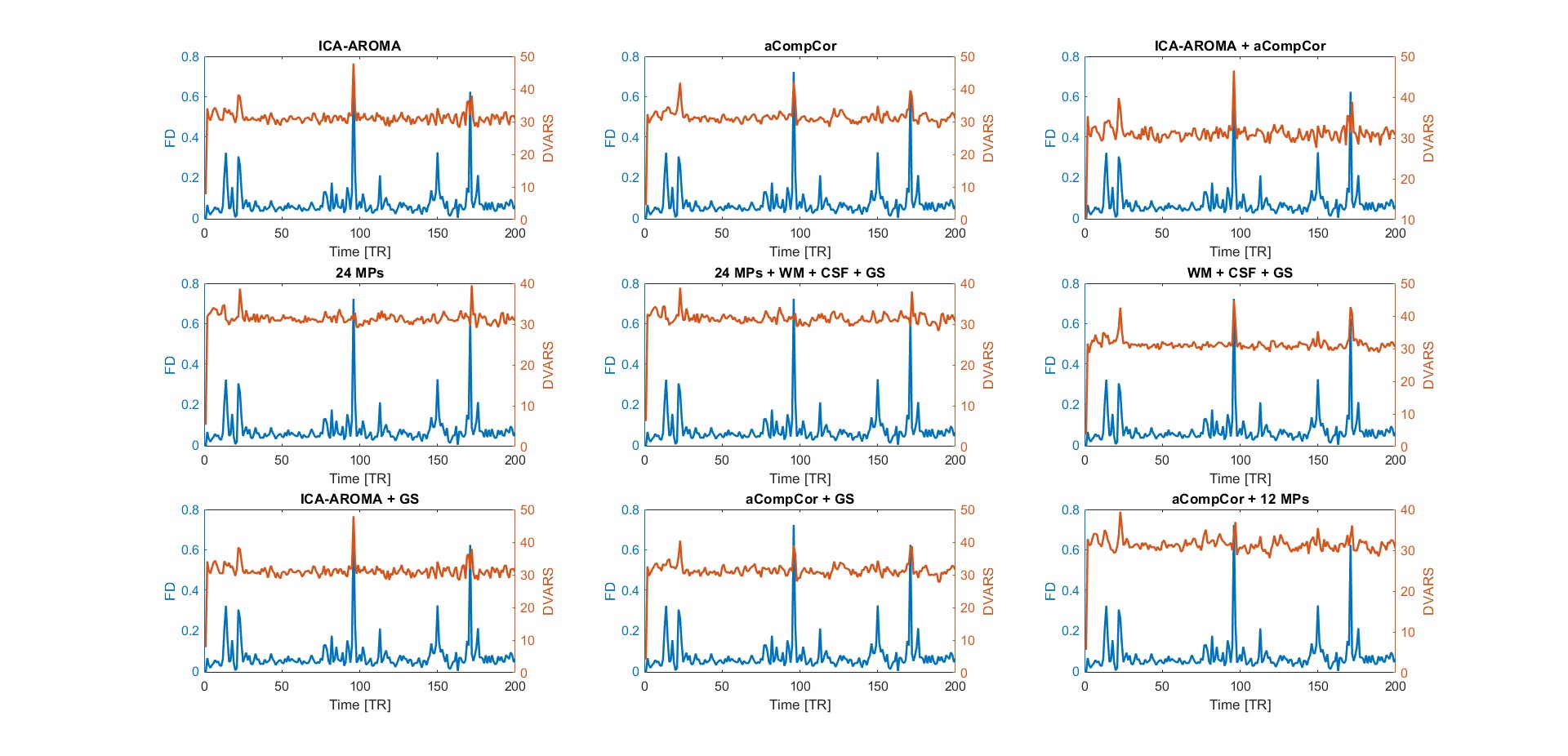


**Fig. S24. FD-DVARS plots for a low-motion subject.** FD and DVARS time series are shown for the minimal preprocessing and for each denoising pipeline.

**FC matrices**

**
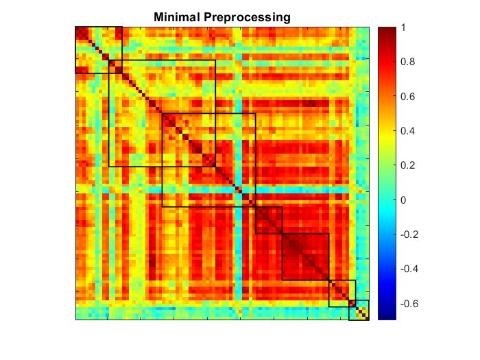
**

**
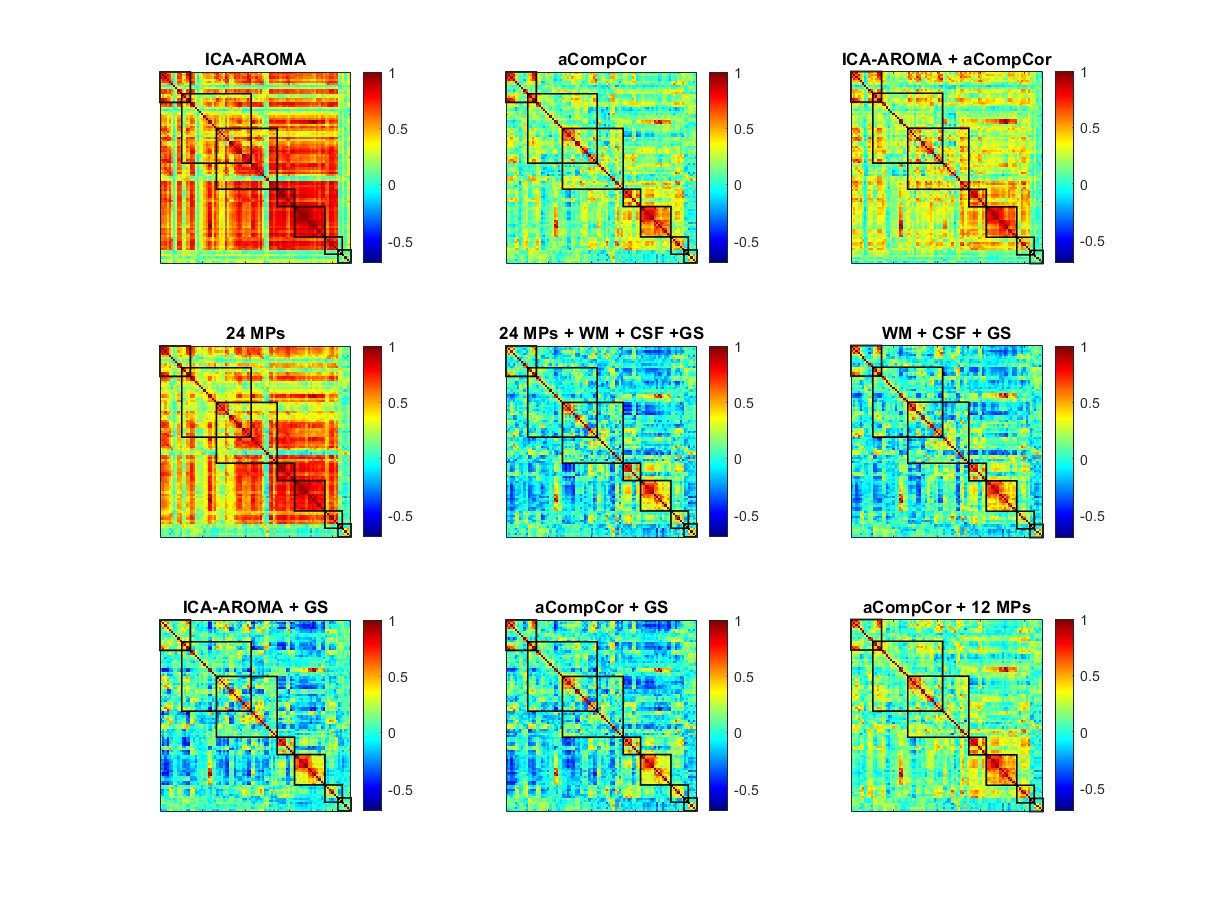
**

**Fig. S25. FC matrices for a high-motion subject.** Pairwise correlation matrices (Peason’s correlation coefficient), with ROIs reorganized in RSNs, are shown for the minimal preprocessing and for each denoising pipeline.

**Wavelet coherence spectra**


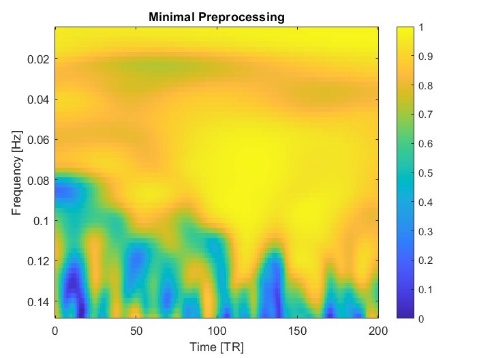


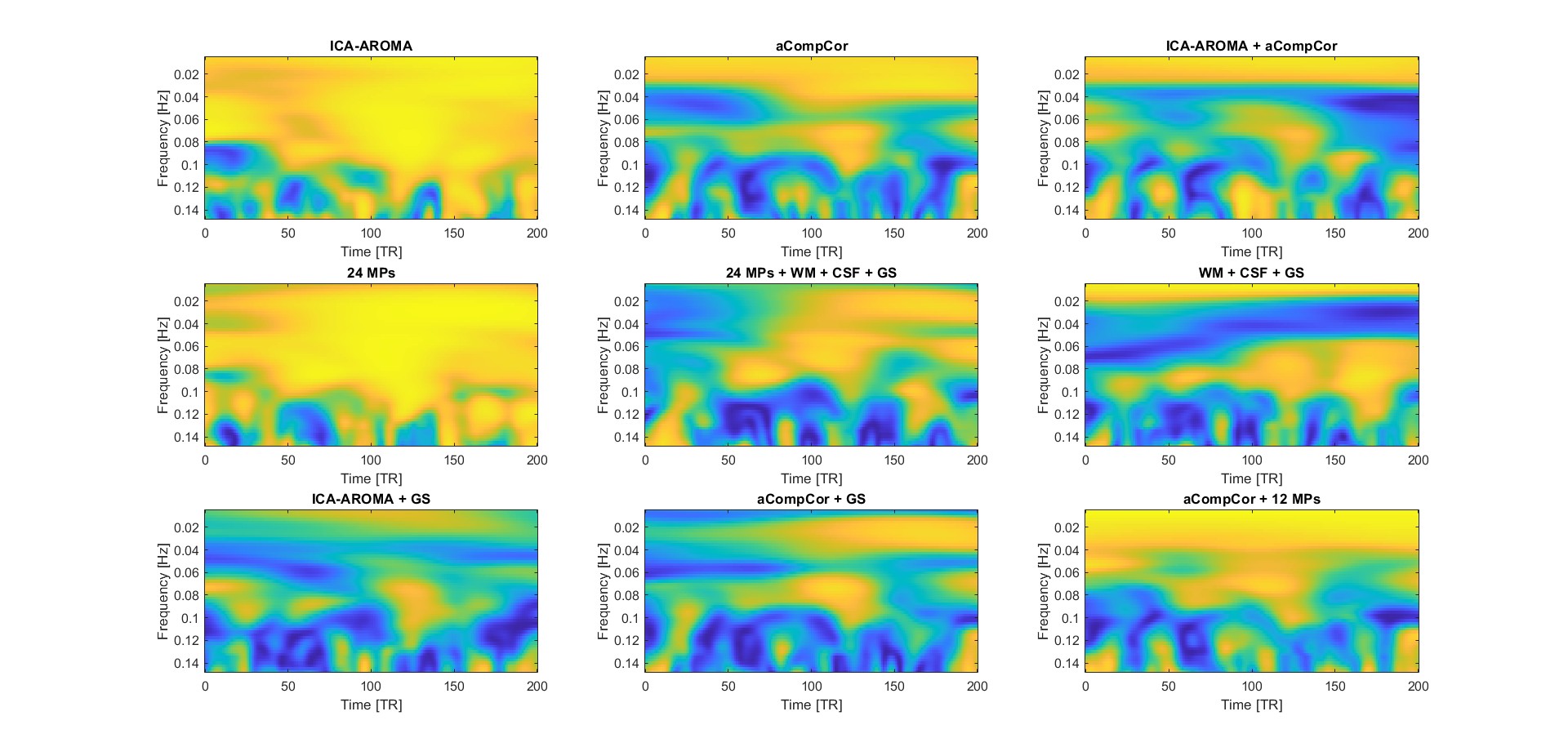


**Fig. S26. Wavelet coherence spectra for a high-motion subject.** Wavelet coherence spectra estimated from the BOLD signals of an exemplar pair of ROIs are shown for the minimal preprocessing and for each denoising pipeline.

**Power spectral densities**

**
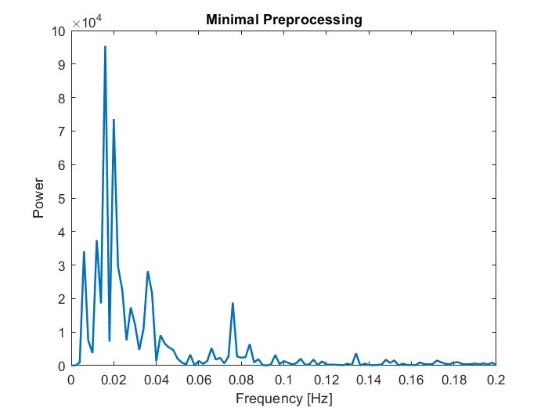
**

**
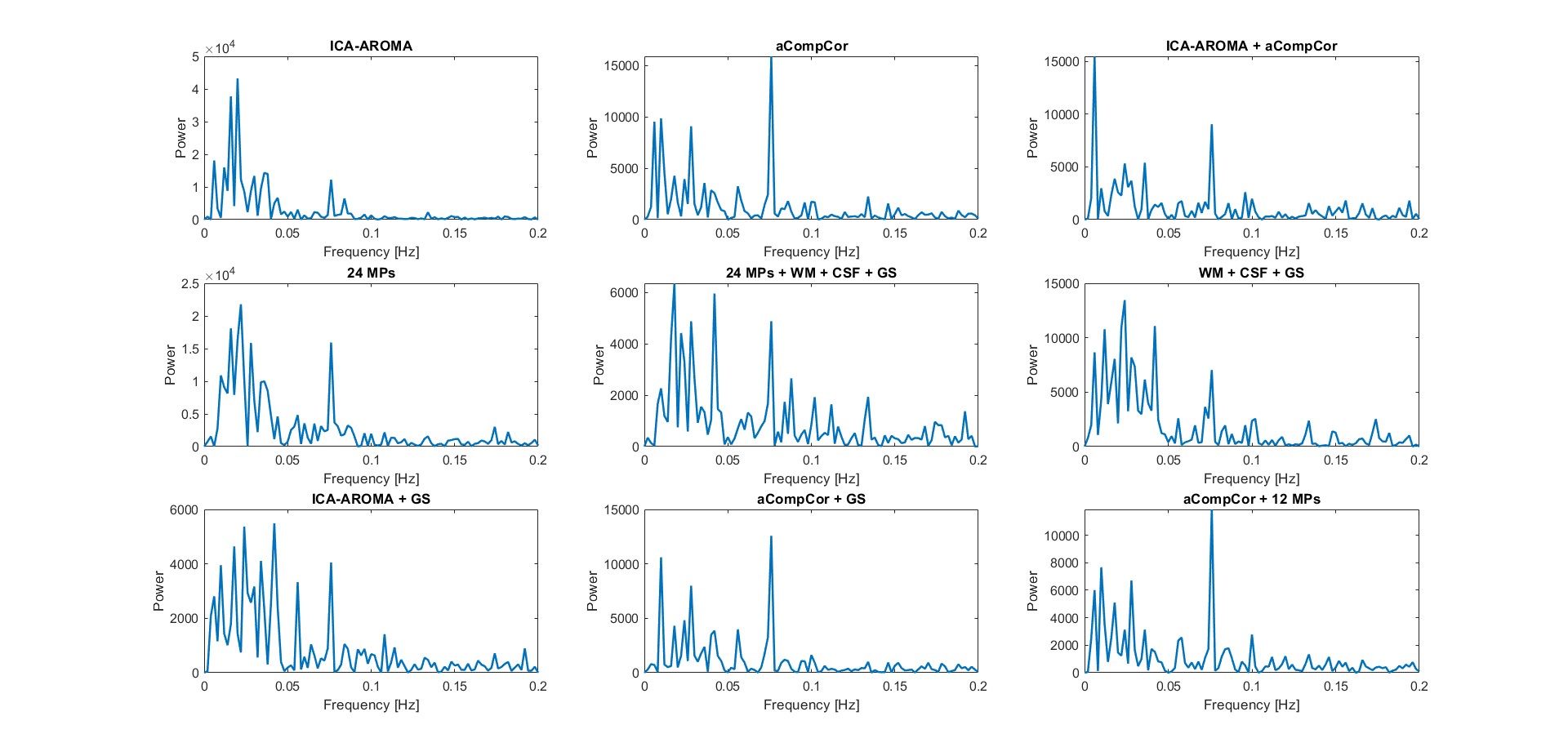
**

**Fig. S27. Power spectra for a high-motion subject.** Power spectral densities estimated from the BOLD signal of an exemplar ROI are shown for the minimal preprocessing and for each denoising pipeline.

1. **Single-network time-based FCC**

**
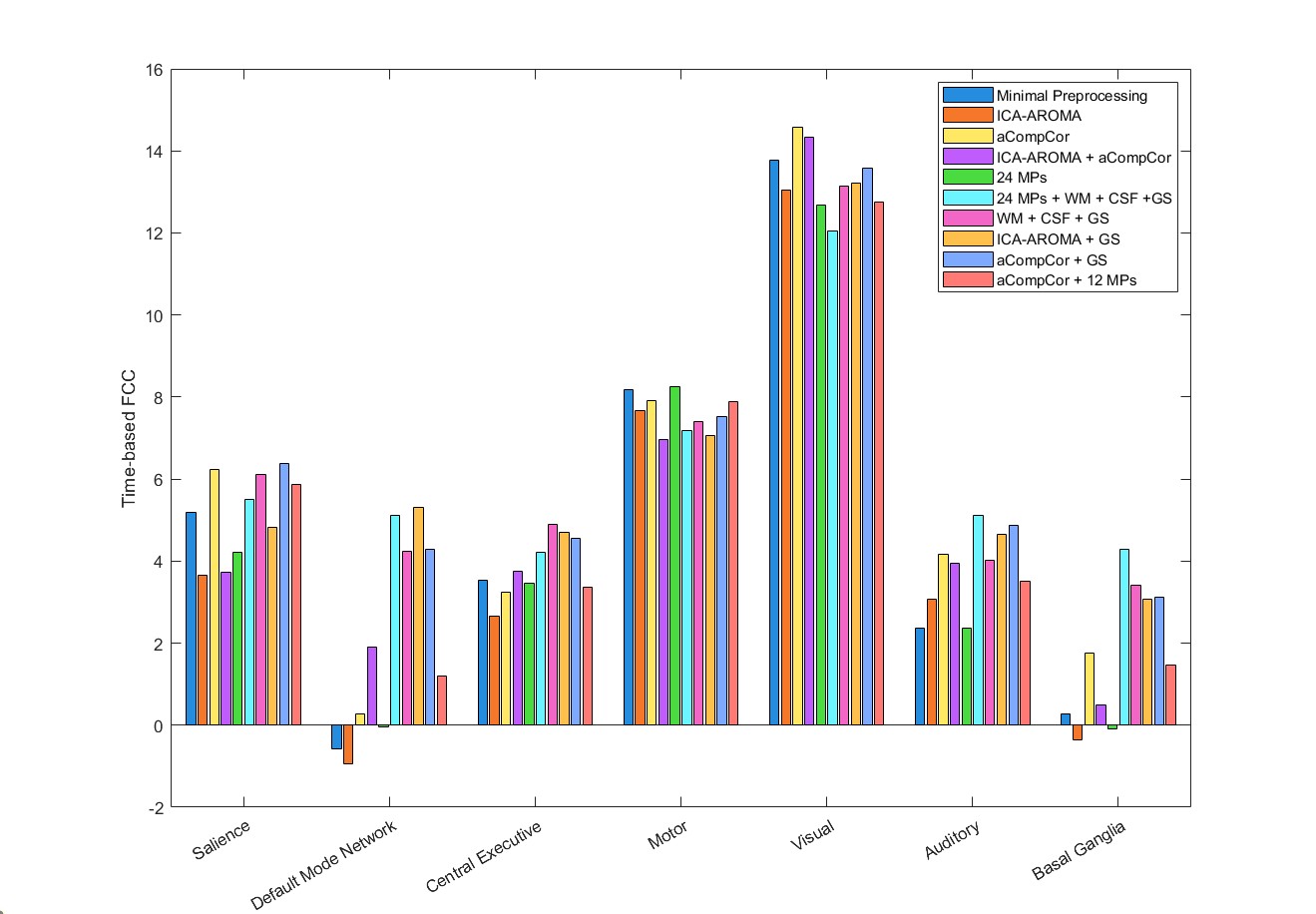
**

**Fig. S28. Single-network time-based FCC.** In the figure, the median time-based FCC values across subjects for each resting state network and each pipeline are plotted.

1. **Simulation pilot study**

**Background**

A simulation pilot study was performed to test the efficacy of the different denoising pipelines on synthetic rs-fMRI data. In the fMRI field, there is a current need to understand how to reliably evaluate advanced methods for collecting and analyzing fMRI data. Indeed, one of the major issues of this kind of data is that they have no ground truth, which is needed to ensure validation of the fMRI analysis methods. Great efforts to establish a gold standard were based on developing mechanical methods or direct measuring the neural activity with intracranial electroencephalogram. However, for most studies, simulation is the only available approach to establish the fMRI ground truth.

So far, most of the fMRI simulation studies used a block or event-related design for the generation of the BOLD activity (Welvaert & Rosseel, 2014), whereas most difficult is the fMRI simulation during resting state since there are no a priori hypotheses on specific brain regions activation and BOLD signal waveform.

In our study, we adopted fmrisim, which is a new Python package for standardized, realistic simulation of fMRI data (Ellis et al., 2020). This package is part of BRAINIAK, “Brain Imaging Analysis Kit, <http://brainiak.org>”, which is a recently released open-source toolbox for advanced neuroimaging analyses (Kumar et al., 2020, 2021). This toolbox leverages advancements from previous fMRI simulators and is specifically tailored for advanced multivariate neuroimaging methods, by employing a linear model that estimates and combines known sources of neural noise. The fmrisim tool is realistic in the sense that it approximates some of the known properties of fMRI data, like simTB (Erhardt et al., 2012) and STANCE (Hill et al., 2017), while also producing entirely novel volumetric that data can be preprocessed with the same tools that are typically used for fMRI analysis, like neuRosim (Welvaert, 2013; Welvaert et al., 2011; Welvaert & Rosseel, 2014).

Since fmrisim simulates BOLD activity through block or event-related designs, for the “ground truth” rs-fMRI signal generation we adopted an independent approach, and finally we combined noise and signal components to achieve the entire 4D resting-state fMRI simulation.

**Materials and methods**

The process of simulating fMRI data consists of three main steps: (i) specifying noise parameters, (ii) generating noise, and (iii) generating signal.

**Parameters setting and noise generation**

The fmrisim tool generates various established sources of fMRI noise and integrates them to produce simulations that exhibit characteristics that are similar to real fMRI data. fmrisim linearly combines a set of noise sources, inspired by biology and MRI physics, that are data-driven tuned (Ellis et al., 2020).

First, to simulate fMRI data using fmrisim, a set of parameters that describe the signal, the noise, and the acquisition setting need to be specified. Some of them can be inferred from real, pre-existing fMRI data and then used to simulate data with similar properties. For this reason, we loaded 4D fMRI data from an exemplar subject that was used as input in the fmrisim pipeline. The size of the fMRI volume and the resolution of the voxels was extracted from real fMRI data.

The simulation toolbox also needs an activity template of the brain that is generated by averaging each voxel of the real fMRI sequence across time (Welvaert et al., 2011) and that is then used to create a binary mask of brain and non-brain voxels, as shown in Fig. S29.


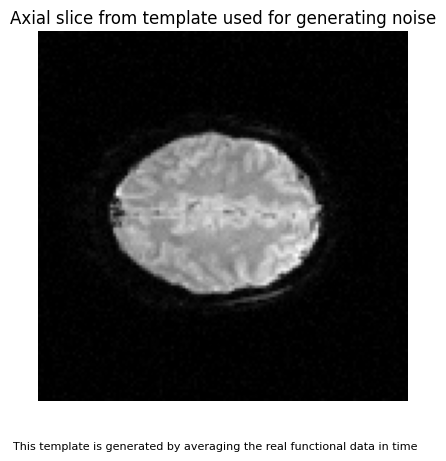

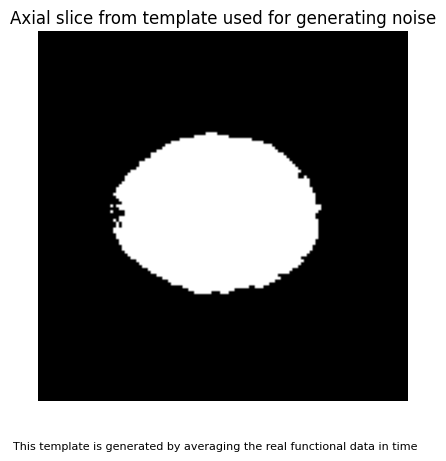


(a) Template. (b) Binary mask.

**Fig. S29.** Axial slice from template (generated by averaging the real fMRI data in time) used for generating noise.

Raw fMRI data are used to estimate the noise parameters that describe general statistics about the noise in the brain. fmrisim can generate various types of realistic fMRI noise, as drift, autoregressive/moving-average (ARMA), physiological, task-related, and system noise. These multiple components are created by receiving the specification of the estimated noise parameters and simulating new whole brain data with noise properties approximating those parameters.

Drift and system noise are assumed to reflect machine-related noise, and consequently affect the entire field of view during the acquisition process. The other components are assumed to be specific of the brain and thus have smoothness components related to the smoothness of functional data.

Drift is simulated by combining cosine basis functions of different phases, with longer runs being comprised of more basis functions.

$$\sum_{i=1}^{L/A} \cos\left( i\pi\frac{t}{L}+p_{i} \right)r^{i-1}$$

where $L$ is the length in seconds of the run, $A$is the volume acquisition time (TR) in seconds, $i$ is the basis function counter, $t$ is the timestamp of each volume in seconds, $p_{i}$ is a random phase in radians for each iteration of $i$, $r$ is the proportion of drop off found by solving $0.99=(1-r^{\frac{2L}{P}})/(1-r^{\frac{2L}{A}})$for $r$, where $P$ is the periodicity.

ARMA noise is generated by creating a sequence of volumes, with each volume being generated by combining Gaussian noise with a specified proportion of the previous volumes (Purdon & Weisskoff, 1998).

$$Y_{t}=X_{t}+\varphi Y_{t-1}+\omega X_{t-1}$$

where $Y$ is the simulated noise at time point $t$, $X$ is the component of new 3D Gaussian noise, $\varphi$ is the AR weight, and $\omega$ is the MA weight.

Physiological noise is modelled by combining sine waves comprised of heart rate (1.17 Hz) and respiration rate (0.2 Hz) (Biswal et al., 1996) with random phase.

Task-related noise, which is simulated in fmrisim by adding Gaussian or Rician noise to time points where there are events (determined by the design of the experiment), was not implemented in our case since we focused on the resting-state condition.

The ARMA and physiological noise components are mixed based on user-defined parameters and adjusted to the appropriate magnitude of temporal variance, determined by the Signal-to-Fluctuation-Noise Ratio (SFNR). The SFNR, also known as temporal signal-to-noise, reflects how much temporal variation there is in brain voxels relative to their mean activity. Drift is weighted separately and added after this.

$$W\left( w_{ARMA}v_{ARMA}+ w_{physio}v_{physio} \right)+ w_{DRIFT}v_{DRIFT}$$

where $W$ is the brain-specific noise that is determined by the standard deviation of the detrended temporal variability, averaged across the brain, $w$ is a weight fit on the data and $v$ is a 4D volume of normalized noise.


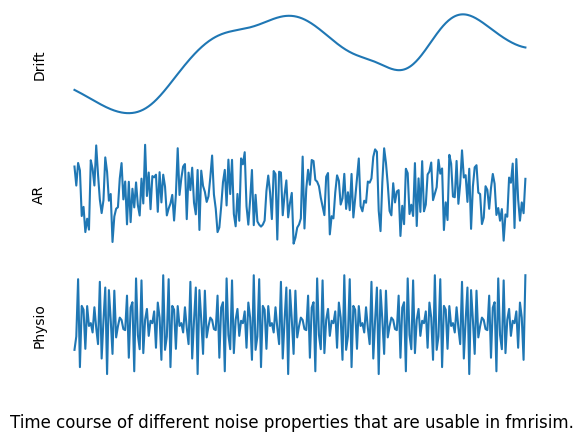


**Fig. S30.** Time course of drift, AR, and physiological noises.

The volume created by the combination of these noise components is then added with system noise, which results from heat-related motion in MRI scans (Bodurka et al., 2007). System noise is approximated by Gaussian noise that then is added to the template of average voxel activity. The magnitude of system noise is determined by both a spatial noise component, which depends on the Signal to Noise Ratio (SNR), and a temporal component of system noise, that depends on the SFNR.

Once the noise parameters are estimated, they are then used to generate time series data to which one or more hypothesized neural signals are added.

Motion has substantial effects on fMRI data quality, but it was not accounted for in this toolbox. Other simulations have modeled the effects of rigid body transformations and motion between slice acquisitions, but these effects can be easily removed with linear motion correction software. The more complex effects of motion are harder to simulate, such as the non-linear changes to T1 relaxation across planes, and are not addressed in fmrisim (Ellis et al., 2020).

**Signal generation**

fmrisim toolbox was used to generate only the noise-related fMRI data, since it accounts for signal generation in task-based block or event-related fMRI designs, whereas our application considered a resting-state protocol.

We generated a synthetic BOLD signal independently in MATLAB environment and then integrate it with noise fMRI data from fmrisim. To simulate BOLD signals, we generated time series mimicking the underlying neuronal activity and convolved them with the canonical hemodynamic response function (HRF).

Specifically, we simulated the BOLD signals corresponding to the voxels of 12 anatomical brain regions belonging to the default mode network (DMN). Each node was defined as a cube composed by 5x5x5 voxels (cube length = 10 mm) centered in the centroid of the region, according to the AAL atlas.

We created a time series of white Gaussian noise of 500 samples, which was used as input to generate a second order AR time series. The latter was then convolved with the canonical HRF to estimate the predicted pattern of measured activity, and then down sampled at real fMRI time points (TR=2.5 seconds) (see Fig. S31).

This signal is what we considered the true signal, our ground truth. We then simulated the signals of all other voxels of the DMN ROIs by adding Gaussian noise to the original signal.

Signal intensity was set to be in the noise range of the same brain region to avoid destroying brain anatomy (by changing gray intensity levels) and preventing not accurate segmentation and normalization steps in the subsequent fMRI pre-elaboration.

We decided to use the same “signal” across all the DMN voxels to have a ground truth for the functional connectivity matrix, and to understand how much the various denoising pipelines that were subsequently parallelly applied were able to preserve it.


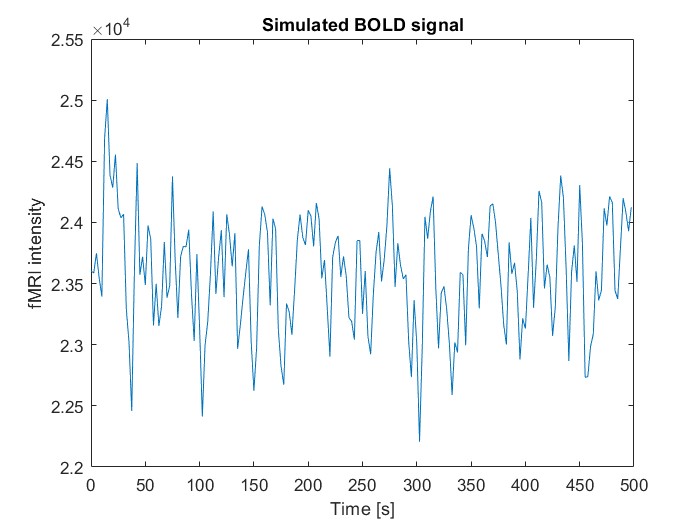


**Fig. S31.** Example of simulated BOLD signal.

**Simulated fMRI data preprocessing and performance evaluation**

The raw simulated fMRI dataset was used as input in HALFpipe as described in section 2.3 and 2.4 in the main text. The real anatomical T1-weighted image of the same subject whose real fMRI data was exploited in fmrisim to compute the brain activity template and mask was used as structural correspondence for preprocessing segmentation and normalization in HALFpipe toolbox.

Finally, the different performance measures and the summary performance index, as described in section 2.5 and 2.6 respectively, were computed on the pre-elaborated fMRI data.

**Results and discussion**

In this section, the simulation study results are presented. The values for all performance measures and summary index are reported in table T.S6.

**Table T.S6. Results of the performance metrics for each denoising pipeline after z-score normalization.**

| Performance metrics | Denoising pipelines | | | | | | | | | |
| --- | --- | --- | --- | --- | --- | --- | --- | --- | --- | --- |
|  | **Baseline** | **ICA-AROMA** | **aCompCor** | **ICA-AROMA + aCompCor** | **24 MPs** | **24 MPs + WM + CSF + GS** | **WM + CSF + GS** | **ICA-AROMA + GS** | **aCompCor + GS** | **aCompCor + 12 MPs** |
| FD-DVARS | -0.671 | -0.258 | -0.588 | -0.207 | 1.856 | 1.883 | -0.678 | -0.272 | -0.589 | -0.475 |
| HF-FC content | -2.546 | 0.673 | 0.153 | 0.768 | 0.209 | 0.334 | -0.821 | 0.676 | 0.189 | 0.364 |
| Modularity | 0.667 | -0.487 | -1.085 | -0.947 | 0.434 | 1.025 | 1.856 | -0.259 | 0.019 | -1.222 |
| Time-based FCC | 1.266 | -1.299 | 0.588 | -1.057 | 0.532 | 0.279 | 0.772 | -1.705 | 0.621 | 0.004 |
| Frequency-based FCC | 0.116 | -1.424 | 1.611 | -1.067 | 0.369 | -0.821 | 0.559 | -0.924 | 0.810 | 0.770 |
| LF BOLD content | 1.603 | -1.212 | 0.395 | -1.347 | 0.217 | 0.007 | 1.151 | -1.230 | 0.312 | 0.104 |
| tDOF loss | 0.944 | -1.324 | 0.699 | -1.568 | 0.359 | 0.285 | 0.871 | -1.349 | 0.676 | 0.407 |
| Summary performance index | 0.116 | -0.710 | 0.234 | -0.734 | **0.589** | **0.472** | **0.442** | -0.684 | 0.259 | 0.017 |

The explorative results obtained on the pilot synthetic rs-fMRI dataset are overall consistent with the results obtained on the real rs-fMRI dataset.

According with FD-DVARS, the MPs regression (24 MPs and 24 MPs + WM + CSF + GS) provided the lower correlation values between FD and DVARS time series, suggesting lower residual variance associated with motion noise. However, in absence of actual motion simulation, MPs parameters are probably acting as random noise regressors that could help to remove unmodeled variance in the data.

Pipelines including ICA-AROMA, followed by MPs regression and aCompCor, confirmed their higher ability in reducing artifacts in fMRI data, as shown by HF-FC content measure.

As regards to network-sensitive measures, higher modularity values were found for WM + CSF + GS and for 24 MPs + WM + CSF + GS. Time-based FCC rewarded the pipelines such as aComCor, WM + CSF + GS, and aCompCor + GS, whereas frequency-based FCC was found to have higher value in all pipelines including aCompCor (aCompCor, aCompCor + GS, and aCompCor + 12 MPs) and WM + CSF + GS. Also on simulated fMRI data, pipelines including GSR showed the higher values in network-sensitive measures, suggesting an enhanced capability in highlighting subnetworks and modules within the whole-brain network.

Considering signal-sensitive metrics, WM + CSF + GS, aCompCor, and aCompCor + GS showed to better preserve fluctuations in the LF range of interest. The pipelines with the lowest number of lost tDOF were the same with the highest values for LF BOLD content.

Finally, the summary performance index showed that the best-performing pipelines were 24 MPs, 24 MPs + WM + CSF + GS, and WM + CSF + GS, followed by pipelines including aCompCor.

However, considering that fmrisim does not simulate motion-related artifacts, the results rewarding the 24 MPs (+ WM + CSF + GS) pipelines should be cautiously interpreted. Instead, even if not much aggressive on artifacts (the worst value in HF-FC content measure), WM + CSF + GS pipeline showed a good trade-off between artifact-reduction, signal of interest preservation and large-scale network enhancement also on synthetic rs-fMRI data.

An additional quality control check was performed considering only the DMN. We compared the functional connectivity matrix computed on the BOLD signals belonging to DMN brain regions for the different denoising pipelines with respect to the minimal preprocessing (see Fig. S32). The mean value of the absolute value of the difference between the pre- and post- functional connectivity matrices is reported in table T.S7.

**Table T.S7. Mean value of the functional connectivity matrices differences.**

|  | Denoising pipelines | | | | | | | | | |
| --- | --- | --- | --- | --- | --- | --- | --- | --- | --- | --- |
|  | **Baseline** | **ICA-AROMA** | **aCompCor** | **ICA-AROMA + aCompCor** | **24 MPs** | **24 MPs + WM + CSF + GS** | **WM + CSF + GS** | **ICA-AROMA + GS** | **aCompCor + GS** | **aCompCor + 12 MPs** |
| Mean difference of DMN FC | - | 0.305 | 0.199 | 0.316 | 0.096 | 0.106 | 0.046 | 0.279 | 0.201 | 0.178 |

In accordance with the summary index results, WM + CSF + GS and the two 24 MPs pipelines were the denoising strategies with the lowest DMN FC differences, suggesting their higher ability to preserve our “ground truth” signal, which is the only time series that was present in these voxels of interest.


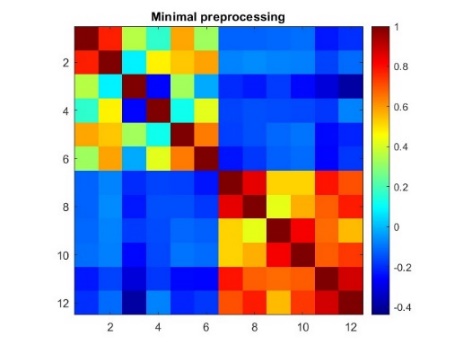


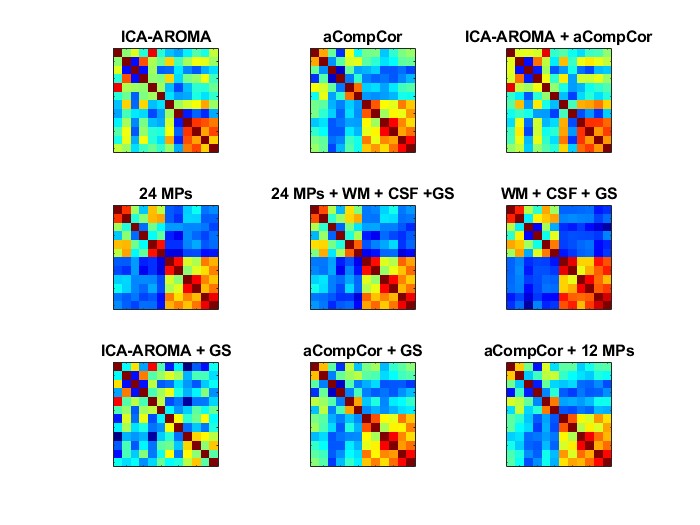


**Fig. S32.** DMN FC matrices computed for the different denoising pipelines.

**References**

Biswal, B., Deyoe, E. A., & Hyde, J. S. (1996). Reduction of physiological fluctuations in fMRI using digital filters. *Magnetic Resonance in Medicine*, *35*(1), 107–113. https://doi.org/10.1002/mrm.1910350114

Bodurka, J., Ye, F., Petridou, N., Murphy, K., & Bandettini, P. A. (2007). Mapping the MRI voxel volume in which thermal noise matches physiological noise—Implications for fMRI. *NeuroImage*, *34*(2), 542–549. https://doi.org/10.1016/j.neuroimage.2006.09.039

Ellis, C. T., Baldassano, C., Schapiro, A. C., Cai, M. B., & Cohen, J. D. (2020). Facilitating open-science with realistic fMRI simulation: Validation and application. *PeerJ*, *8*, e8564. https://doi.org/10.7717/peerj.8564

Erhardt, E. B., Allen, E. A., Wei, Y., Eichele, T., & Calhoun, V. D. (2012). SimTB, a simulation toolbox for fMRI data under a model of spatiotemporal separability. *NeuroImage*, *59*(4), 4160–4167. https://doi.org/10.1016/j.neuroimage.2011.11.088

Hill, J. E., Liu, X., Nutter, B., & Mitra, S. (2017). A task-related and resting state realistic fMRI simulator for fMRI data validation. *Medical Imaging 2017: Image Processing*, *10133*, 748–759. https://doi.org/10.1117/12.2254777

Kumar, M., Anderson, M. J., Antony, J. W., Baldassano, C., Brooks, P. P., Cai, M. B., Chen, P.-H. C., Ellis, C. T., Henselman-Petrusek, G., Huberdeau, D., Hutchinson, J. B., Li, Y. P., Lu, Q., Manning, J. R., Mennen, A. C., Nastase, S. A., Richard, H., Schapiro, A. C., Schuck, N. W., … Norman, K. A. (2021). BrainIAK: The Brain Imaging Analysis Kit. *Aperture Neuro*, *1*(4). https://doi.org/10.52294/31bb5b68-2184-411b-8c00-a1dacb61e1da

Kumar, M., Ellis, C. T., Lu, Q., Zhang, H., Capotă, M., Willke, T. L., Ramadge, P. J., Turk-Browne, N. B., & Norman, K. A. (2020). BrainIAK tutorials: User-friendly learning materials for advanced fMRI analysis. *PLoS Computational Biology*, *16*(1), e1007549. https://doi.org/10.1371/journal.pcbi.1007549

Purdon, P. L., & Weisskoff, R. M. (1998). Effect of temporal autocorrelation due to physiological noise and stimulus paradigm on voxel-level false-positive rates in fMRI. *Human Brain Mapping*, *6*(4), 239–249. https://doi.org/10.1002/(SICI)1097-0193(1998)6:4&#x0003c;239::AID-HBM4&#x0003e;3.0.CO;2-4

Welvaert, M. (2013). *Simulation of fMRI data: A statistical approach*. https://www.semanticscholar.org/paper/Simulation-of-fMRI-data%3A-a-statistical-approach-Welvaert/89cb15e9c9d9cfe58b5d07d51c16f453912e3155

Welvaert, M., Durnez, J., Moerkerke, B., Berdoolaege, G., & Rosseel, Y. (2011). neuRosim: An R Package for Generating fMRI Data. *Journal of Statistical Software*, *44*, 1–18. https://doi.org/10.18637/jss.v044.i10

Welvaert, M., & Rosseel, Y. (2014). A review of fMRI simulation studies. *PloS One*, *9*(7), e101953. https://doi.org/10.1371/journal.pone.0101953
